# Involvement of a eukaryotic-like ubiquitin-related modifier in the proteasome pathway of the archaeon *Sulfolobus acidocaldarius*

**DOI:** 10.1038/ncomms9163

**Published:** 2015-09-08

**Authors:** Rana S. Anjum, Sian M. Bray, John K. Blackwood, Mairi L. Kilkenny, Matthew A. Coelho, Benjamin M. Foster, Shurong Li, Julie A. Howard, Luca Pellegrini, Sonja-Verena Albers, Michael J. Deery, Nicholas P. Robinson

**Affiliations:** 1 Department of Biochemistry, University of Cambridge, Tennis Court Road, Cambridge CB2 1GA, UK; 2 Department of Biochemistry and Cambridge Systems Biology Centre, Cambridge Centre for Proteomics, Cambridge CB2 1QR, UK; 3 Molecular Biology of Archaea, Max Planck Institute for Terrestrial Microbiology, 35043 Marburg, Germany

## Abstract

In eukaryotes, the covalent attachment of ubiquitin chains directs substrates to the proteasome for degradation. Recently, ubiquitin-like modifications have also been described in the archaeal domain of life. It has subsequently been hypothesized that ubiquitin-like proteasomal degradation might also operate in these microbes, since all archaeal species utilize homologues of the eukaryotic proteasome. Here we perform a structural and biochemical analysis of a ubiquitin-like modification pathway in the archaeon *Sulfolobus acidocaldarius.* We reveal that this modifier is homologous to the eukaryotic ubiquitin-related modifier Urm1, considered to be a close evolutionary relative of the progenitor of all ubiquitin-like proteins. Furthermore we demonstrate that urmylated substrates are recognized and processed by the archaeal proteasome, by virtue of a direct interaction with the modifier. Thus, the regulation of protein stability by Urm1 and the proteasome in archaea is likely representative of an ancient pathway from which eukaryotic ubiquitin-mediated proteolysis has evolved.

## Abstract (web summary)

Small archaeal ubiquitin-like modifiers (SAMPs) have been hypothesized to be part of an ancestral version of the ubiquitin-proteasome system. Here, Anjum *et al.* identify a SAMP homologous to the eukaryotic ubiquitin-related modifier-1 and show that it is processed by the 20S core proteasome in *S. acidocaldarius*.

## Graphical abstract

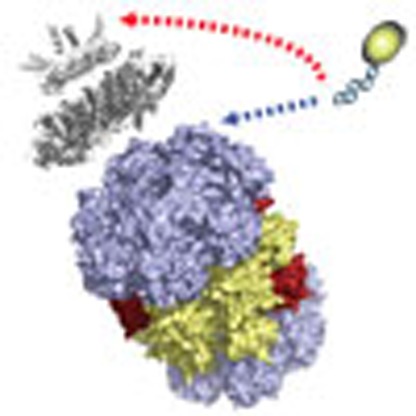

Ubiquitin (Ub) and the related ubiquitin-like proteins (Ubls), belong to a family of modifiers that covalently attach to a diverse array of cellular targets, and orchestrate a wide variety of regulatory processes in the eukaryotic cell. The role of ubiquitylation (also referred to as ubiquitination) in proteasome-mediated proteolysis pathways has been well characterised^1^,^2^. In addition to the roles in protein turnover, Ub and Ubl conjugation also operates as a post-translational signal to regulate diverse cellular processes and pathways including DNA replication, DNA damage repair, transcription, cell-cycle control, chromatin modification, protein trafficking, autophagy and innate immunity^3^,^4^,^5^,^6^,^7^.

Ubls were originally thought to be confined to the eukaryotic domain of life. However, the discovery of an unanticipated structural homology between the bacterial sulphur-transfer proteins ThiS and MoaD, and the eukaryotic Ub/Ubl proteins^8^,^9^ led to speculation that primitive prokaryotic homologues are the antecedents of the eukaryotic Ub/Ubl family^10^. These prokaryotic ubiquitin-like homologues share the characteristic Ub/Ubl-like β-grasp structural fold^11^,^12^,^13^, and are activated with similar catalytic chemistry to Ub/Ubls^10^. However, their primary biological role is to mobilize sulphur during biosynthetic reactions^8^,^9^,^14^,^15^.

The eukaryotic Urm1 (ubiquitin-related modifier-1) protein, which displays both structural and amino-acid sequence homology to the prokaryotic ThiS and MoaD, has been proposed as a candidate for the evolutionary ‘missing link' between the eukaryotic Ub/Ubl family and the ancestral prokaryotic sulphur-transfer proteins^16^,^17^,^18^,^19^,^20^,^21^,^22^,^23^. Although ubiquitylation reactions proceed via three well-characterised enzymatic steps, known as the E1-E2-E3 cascade^24^, urmylation events are apparently less complex and only require an E1-like enzyme to generate covalently modified substrates^21^,^22^. In addition, Urm1 also mediates sulphur mobilization reactions during post-translational tRNA modification reactions^25^,^26^. This functional duality led to further speculation that some prokaryotic sulphur-transfer Ubl homologues might also play roles in substrate conjugation.

*Bona fide* ubiquitin-like modifications were eventually identified beyond the eukaryotic domain with the discovery of the SAMP (small Ubl archaeal modifier protein) moieties^27^,^28^,^29^. Covalent attachment of the modifier, referred to as SAMPylation, occurs between the conserved C-terminal di-glycine motif of SAMP, and specific lysine residues on a variety of target proteins, in a manner reminiscent of urmylation and ubiquitylation^27^,^28^,^29^. It has been revealed that the E1-like SAMP activators^30^ adenylate the terminal glycine of the modifier before the covalent attachment to the substrate^30^. Furthermore, the *Haloferax volcanii* and *Methanosarcina acetivorans* SAMP crystal structures confirmed the predicted Ub/Ubl-like β-grasp fold^30^,^31^,^32^. Originally identified in the halophilic archaeon *H. volcanii*, bioinformatic analyses have since indicated that SAMPs are widely distributed across all the archaeal kingdoms^33^.

By comparison, bacteria of the *Actinobacteria* and *Nitrospirae* families employ an analogous covalent modification system referred to as pupylation^34^,^35^. These bacterial species unusually possess homologues of the eukaryotic/archaeal type proteasomal machinery^36^,^37^, in addition to the more commonly distributed bacterial proteolytic apparatus^38^,^39^,^40^,^41^. Attachment of the modifier Pup (prokaryotic ubiquitin-like protein) directs substrate delivery to the proteasome, but unlike ubiquitin-mediated proteolysis the Pup tag can also be destroyed along with the target^42^. Bacterial Pup does not belong to the β-grasp family^43^, however, and has presumably evolved independently from the eukaryotic Ub and archaeal SAMP proteins.

Although the eukaryotic ubiquitylation and archaeal SAMPylation pathways have evolved from a common antecedent, and despite previous studies suggesting a link between SAMPylation and proteasomal degradation^27^,^28^,^33^, direct evidence of an interaction between the modifier and the proteasome is still lacking^44^. To investigate the possible crosstalk between SAMPylated substrates and the archaeal proteasome, we investigated these processes in the biochemically tractable thermophilic archaeon *Sulfolobus acidocaldarius*. Here we solve the crystal structure of an Urm1/SAMP homologue from the related species *S. solfataricus*, which reveals similarities to both eukaryotic Urm1 proteins, and other archaeal SAMPs. Using tandem mass-spectrometry, we demonstrate that this modifier is conjugated to a variety of targets both *in vivo* and *in vitro*. Furthermore, we demonstrated that modified substrates are recognized directly by the *S. acidocaldarius* 20S core proteasome, and by the proteasome-activating nucleotidase (PAN) ATPase^44^. Taken together, our data suggest that Urm1/SAMP modification acts as a signal for substrate recognition by the archaeal proteasome. We discuss our findings in relation to the evolution of Urm1/SAMPs, other Ub/Ubl modifiers, and proteasomal targeting systems.

## Results

### Crystal structure of an Urm1 homologue in *S. solfataricus*

We initially searched for eukaryotic-like Ub and Ubl homologues in the genomes of the thermophilic archaea *S. acidocaldarius* and *S. solfataricus*. A eukaryotic Urm1 homologue was identified in both crenarchaeal species by BLAST searches (Supplementary Fig. 1), in agreement with an earlier bioinformatics study^33^. Although Urm1 also shares amino-acid sequence homology with both the *Escherichia coli* ThiS and MoaD sulphur transfer proteins^16^, PHYRE2 homology searches revealed that these archaeal Urm1 proteins appear more closely related to eukaryotic counterparts, than to the bacterial ThiS/MoaD sulphur transfer components (Supplementary Table 1).

**Figure 1:**
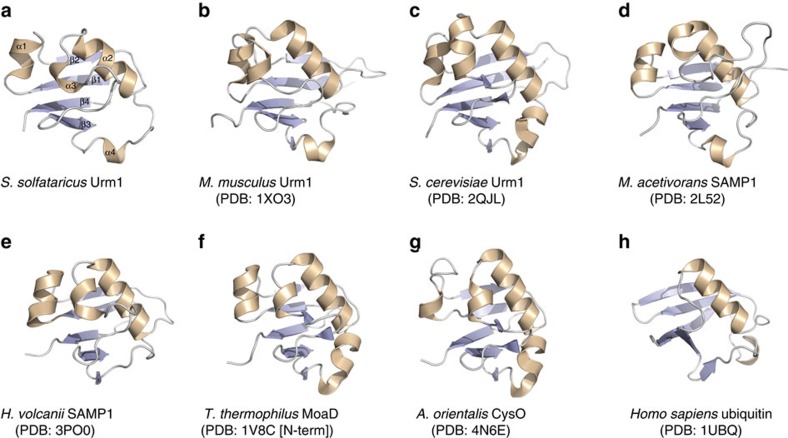
Crystal structure of the *S. solfataricus* Urm1 protein, and comparison with β-grasp fold structural homologues. (**a**) *S. solfataricus* Urm1 structure shown as a ribbon, and coloured according to secondary structure elements; loops are coloured grey, α-helices are shown in wheat, while the β-sheet is depicted in pale blue (all four α-helices and all four β-strands are numbered). (**b**–**g**) Ribbon representation of the *M. musculus* Urm1, *S. cerevisiae* Urm1, *M. acetivorans* SAMP1, *H. volcanii* SAMP1, *T. thermophilus* MoaD and *A. orientalis* CysO β-grasp fold homologues, respectively; all homologues were identified by DALI and VAST searches. (**h**) Ribbon representation of *H. sapiens* ubiquitin. All panels are coloured as described in (**a**). Figures generated using PyMOL^68^.

We substantiated our comparison of the archaeal Urm1/SAMP and eukaryotic Urm1 family proteins by determining the crystal structure of *S. solfataricus* Urm1 at 2.2 Å resolution (Fig. 1a and Supplementary Fig. 2; also see Table 1 for refinement and model statistics). Dali^45^ and VAST^46^ fold recognition searches of the *S. solfataricus* Urm1 structure revealed homology to a number of eukaryotic Urm1 homologues, as well as to the archaeal *M. acetivorans* and *H. volcanii* SAMP1 proteins (Supplementary Table 2; Fig. 1a–e). Additionally, homology was found to *T. thermophilum* MoaD^18^, and the bacterial sulphur transfer protein CysO from *Mycobacterium tuberculosis* and *Amycolatopsis orientalis* (Supplementary Table 2). Unlike other bacterial MoaD/ThiS family homologues, the *T. thermophilum*, and *A. orientalis* proteins displayed exactly the same connectivity of secondary structural elements as the eukaryotic/archaeal Urm1/SAMP modifiers (Fig. 1f,g).

As predicted, the *S. solfataricus* Urm1 adopts a ubiquitin-like β-grasp fold (Fig. 1h), formed from a four-stranded β-sheet packed against a central α-helix (Fig. 1a). Similar to Ub and other Ubls, the β-strands are organized in the order β2-β1-β4-β3. The inner surface of the sheet, facing the α-helix, harbours several buried residues that form the hydrophobic core of the β-grasp fold. Four α-helices are also evident, with the first connecting strands β1 and β2, the second and third located between strands β2 and β3, and the final helix positioned on a long loop between strands β3 and β4 (Fig. 1a). This order of secondary structural features is also conserved in eukaryotic Urm1 proteins, and in the *M. acetivorans* SAMP1 structure (PDB:2L52)^30^ (Fig. 1a–d). The bacterial MoaD family of proteins, and the *H. volcanii* SAMP1 fold (PDB:3PO0)^31^, also display the same order of secondary structural elements, up to the final α-helix, which is often absent (Fig. 1e)^13^,^47^. Interestingly, this final helix, linking the last two β-strands, is a feature shared by the Ub/Ubl and ThiS families^13^.

**Figure 2:**
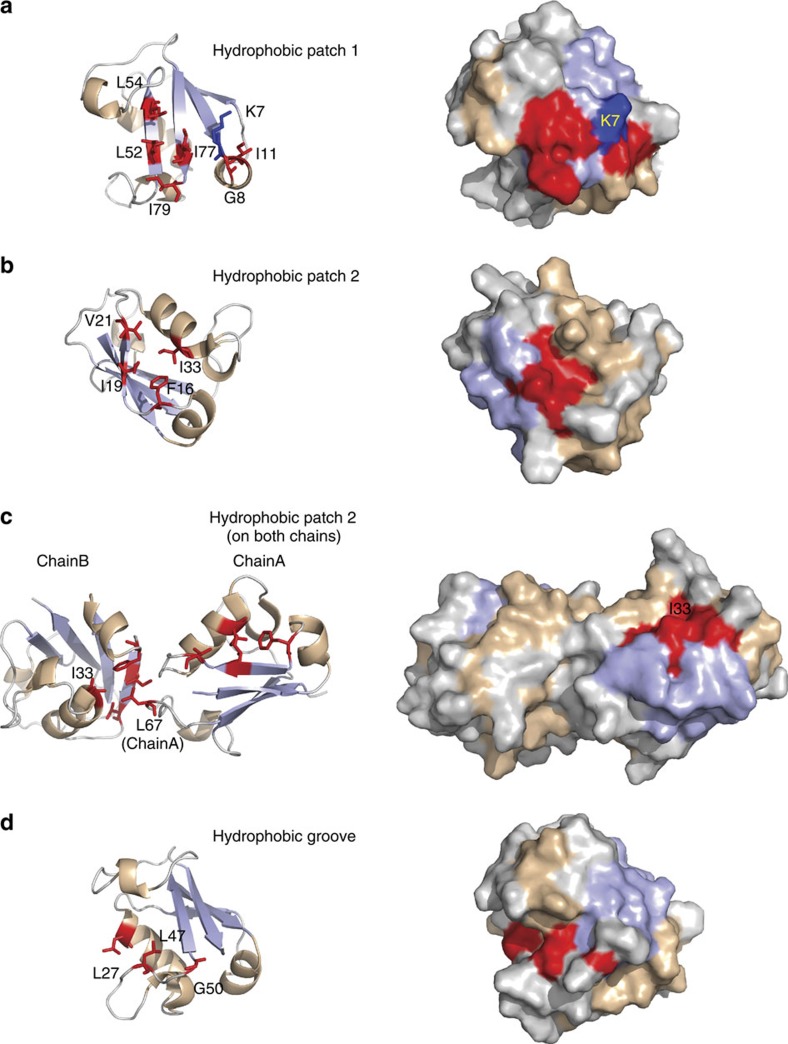
Architecture of *S. solfataricus* Urm1, identifying exposed hydrophobic surfaces and dimerization interfaces. (**a**) Left; *S. solfataricus* Urm1, showing the exposed residues (red sticks) that form the C-terminal exposed hydrophobic patch. The conserved K7 lysine is also shown in dark blue. Right; surface view, identifying the exposed hydrophobic patch 1, coloured in red. (**b**) Left. view from the opposing edge of the β-sheet, on Urm1 chain B, revealing a second hydrophobic patch composed of four exposed residues (F16, I19, V21 and I33; red sticks). *Right*. Surface view displaying the hydrophobic patch 2, in red. (**c**) *Left*: view of Urm1 chains A and B, showing the F16, I19, V21 and I33 patch on both chains. The L67 residue of chain A protrudes into the I33 centred hydrophobic patch on chain B. *Right*. Surface view of the Urm1 dimer, showing the location of the surface exposed I33 residue on chain A. (**d**) Left: a hydrophobic groove, lined with the residues G50, L47 and L27 (red sticks) lies adjacent to the C-terminal exposed hydrophobic patch. Right. Surface view of the C-terminal groove. In all panels secondary structure elements are coloured as in Fig. 1 (loops in grey, α-helices in wheat, and the β-sheet in pale blue). Figures generated using PyMOL^68^.

Inspection of the surface hydrophobicity of the *S. solfataricus* Urm1 structure revealed the presence of a solvent-exposed hydrophobic patch in close proximity to the C-terminal tail (Fig. 2a). This surface is composed of the conserved hydrophobic residues L52 and L54 on strand β3, I77 on strand β4, and I79 on the C terminus of the protein. The conserved residues G8 and I11 also contribute to an adjacent hydrophobic surface, although the K7 lysine residue, conserved across all of the *Sulfolobus* species, bisects these two solvent-exposed hydrophobic regions (Fig. 2a). Equivalent hydrophobic patches have been described previously in ubiquitin, Urm1 and MoaD, where these surfaces are essential for the interaction with partner proteins, including their cognate E1 activating enzyme^3^,^18^,^47^.

A second exposed hydrophobic surface is also present in *S. solfataricus* Urm1, formed by the residues I19 and V21 on strand β2, I33 at the end of helix α2, and F16, on the loop adjoining helix α1 and strand β2 (Fig. 2b). Notably the I33 residue, conserved in the *Sulfolobales* family, was somewhat sunken, creating a shallow hydrophobic depression. This second shallow hydrophobic surface is separate from the first hydrophobic patch, which is located on the opposite face of the central β-sheet (Fig. 2b). We observed in the structure that a leucine (L67) on the final loop on one Urm1 subunit protrudes into the hydrophobic depression of a neighbouring Urm1 chain to form a dimerization interface (Fig. 2c). However, this arrangement may simply be an artefact as a result of packing in the crystal lattice.

The structure also reveals a narrow hydrophobic channel, juxtaposed to the first hydrophobic patch abutting the C-terminal tail. This trough is lined with the surface exposed conserved residues L27, L47 and G50, and extends from the base of the C-terminal tail to the opposite end of the structure (Fig. 2d). The conserved residues W60 and R61, on helix α4, form one of the sidewalls of this groove, separating it from the nearby hydrophobic patch 1. Interestingly, a similar groove has been reported previously in the *M. acetivorans* SAMP1 structure (PDB:2L52)^30^, and this feature can also be observed in the eukaryotic Urm1 protein structures (PDB:2AX5)^18^, but is absent from the bacterial MoaD-like family of proteins (PDB:1JW9)^47^. A functional role for this groove has yet to be ascribed in eukaryotic and archaeal Urm1/SAMP homologues.

### The Urm1 modifier is activated by an ELSA homologue

All eukaryotic Ub and Ubl proteins must be bound and activated by a cognate adenylating enzyme, often referred to as the E1 enzyme. This hydrolyses ATP and catalyses the adenylation of the terminal glycine of the modifier, before substrate conjugation^24^. In the case of the ancestral MoaD/ThiS sulphur carriers, this step is also necessary for sulphur transfer, and is performed by the MoeB and ThiF E1-like enzymes, respectively^14^,^15^. Archaeal E1-like/ELSA homologues have been identified previously in both *M. acetivorans* and *H. volcanii* species^29^,^30^,^48^. We identified a single ELSA homologue (Saci0179) in the *S. acidocaldarius* genome. This displayed approximately equal sequence identity to both the bacterial ThiF enzyme and also to the eukaryotic Uba4p protein, the E1-like homologue for the eukaryotic urmylation pathway. However, a C-terminal rhodanese domain, harboured in eukaryotic E1 enzymes, was absent in the *S. acidocaldarius* enzyme, as has been reported previously for other archaeal and bacterial E1-like proteins^29^,^30^,^48^.

**Figure 3:**
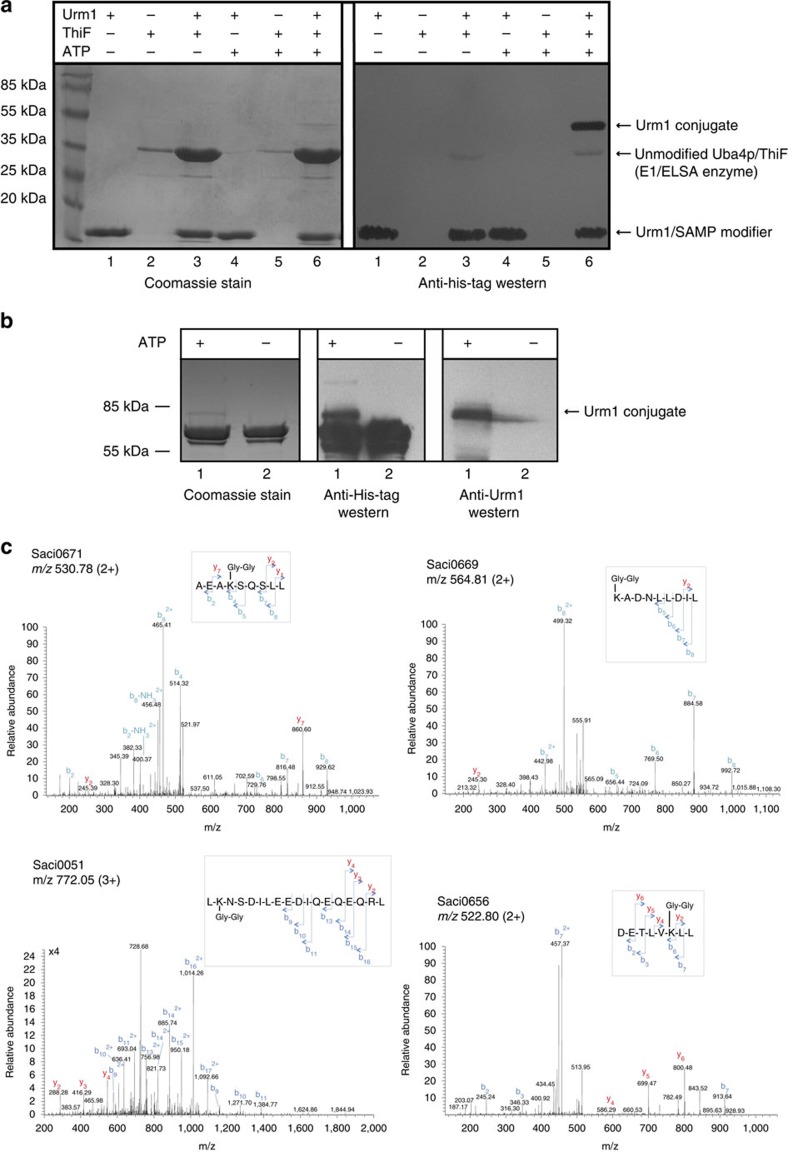
Production of covalent ATP-dependent Urm1 conjugates by the ELSA/Uba4p/ThiF E1-like enzyme. (**a**) Auto-urmylation of the ELSA/Uba4p/ThiF enzyme. 30 μg N-terminally His-tagged Urm1 protein and 90 μg ELSA/Uba4p/ThiF enzyme were incubated together at 70 °C for 1 h in either the presence or absence of 2.5 mM ATP, and retrieved by Ni-NTA agarose pulldown. Left; Coomassie stained gel of the pull-down. Right; western blot of a duplicate gel, probed with an anti-His antibody. Lanes 1 and 4: Urm1 only controls, without or with ATP, respectively; lanes 2 and 5: untagged ELSA/Uba4p/ThiF only controls, without or with ATP, respectively; lanes 3 and 6: Urm1 plus ELSA/Uba4p/ThiF incubations, without or with ATP, respectively. (**b**) Urmylation of the Saci0666 thermosome subunit. 30 μg N-terminally His-tagged Urm1 protein plus 15 μg ELSA/Uba4p/ThiF enzyme and 75 μg C-terminally His-tagged Saci0666 were incubated together and pulled-down as described in (**a**). Lane 1: reaction in the presence of 2.5 mM ATP; lane 2: no ATP control. Left; Coomassie stained gel of the pull-down. Middle and Right; western blot of duplicate gels, probed with an anti-His, or anti-Urm1 antibodies, respectively. (**c**) Example MS/MS spectra of the diglycine modified peptides from the *in vitro* urmylation assay (Saci0671 (FBP) and Saci0669 (Urm1)) and *in vivo* Urm1 overexpression (Saci0051 [Rad50] and Saci0656 [PAN]). *m/z* values of the precursor ions are shown in the top left of each panel. (2+) or (3+) indicates doubly or triply charged precursor ions, respectively. Spectra show the annotated peaks that are due to C-terminal y (coloured red) and N-terminal b (coloured blue) fragment ions. In each case, the *m/z* values of the precursor ions and the *m/z* values of the fragment ions are consistent with diglycine modified lysine residues. The amino-acid sequence of the chymotrypsin-generated peptide, including the di-glycine modified lysine, is shown within the grey box in each example. Other examples are shown in Supplementary Fig. 5.

To test whether the ELSA/Uba4p/ThiF homologue was indeed the E1 activating enzyme, we purified untagged ELSA/Uba4p/ThiF, and an N-terminally His-tagged Urm1; both *S. acidocaldarius* proteins were expressed heterologously in *E. coli*. In order to promote complex formation, the two proteins were then incubated together, in either the presence or absence of ATP, before the potential interaction was examined by pull-down assay (Fig. 3a). We observed the untagged ELSA/Uba4p/ThiF associating with the tagged Urm1 protein independently of the addition of ATP. However, the inclusion of the nucleotide resulted in the formation of a conjugate between the two components. Importantly, this conjugate survived boiling in the presence of reducing agent and SDS, indicative of a covalent attachment (Fig. 3a; lane 6). The conjugate observed here is consistent with the previously reported covalent modification of SAMPs in other archaea mediated by the ELSA, following adenylation of the modifier^27^,^29^,^30^.

Having demonstrated that the ELSA/Uba4p/ThiF homologue was competent to activate the Urm1 protein, ultimately resulting in the auto-urmylation of the E1-like enzyme, we next examined the ability of the complex to modify an exogenous target protein. Given the strong genomic linkage observed between Urm1 and the β–subunit of the thermosome (Saci0666) (see Supplementary note and Supplementary Fig. 3), we purified this chaperonin component in *E. coli* and included the protein in the *in vitro* Urm1 activation reaction. Addition of this protein in the assay resulted in the formation of a clear ATP-dependent covalent conjugate, confirming that the ELSA/Urm1 complex can modify exogenously added substrates *in vitro* (Fig. 3b).

### Mass spectrometry identification of urmylated substrates

It has been established that Ub, eukaryotic Urm1, and archaeal SAMP conjugates are formed between the C-terminal glycine of the modifier and the ɛ-amino-group of acceptor lysine residues on the target proteins^1^,^2^,^20^,^24^,^44^. A mass spectrometry (MS) approach was therefore adopted to asses whether *S. acidocaldarius* Urm1 is also covalently attached to substrates by the same linkage. *S. acidocaldarius* cell-free extract was subjected to an urmylation reaction, using His-tagged Urm1 and untagged ELSA/Uba4p/ThiF enzyme, in the presence of ATP. The resultant His-tagged conjugates were retrieved by pull-down, separated by reducing SDS–polyacrylamide gel electrophoresis (SDS–PAGE), and subsequently digested in-gel with chymotrypsin, before analysis by tandem mass spectrometry (GeLC-MS/MS) (Supplementary Fig. 4A). In parallel to this *in vitro* approach, we also overexpressed His-tagged Urm1 protein from an exogenous expression vector in *S. acidocaldarius* cells. The *in vivo* generated Urm1 conjugates were collected by pull-down following cell disruption, and the resulting material was also analysed by GeLC-MS/MS (Supplementary Fig. 4B). Chymotrypsin treatment resulted in the hydrolysis of the *S. acidocaldarius* Urm1 peptide backbone between the H82 residue and the penultimate G83 residue of a C-terminal di-glycine motif (G83G84). Hence, if Urm1 was conjugated to a target protein, the chymotrypsin treatment resulted in a fragment harbouring the di-glycine motif, branching from a lysine residue on the linear peptide backbone of the substrate. The addition of a di-glycine motif does not increase the size of the peptides beyond the detection limits of the mass spectrometer, thereby facilitating identification of conjugates.

Analysis of both the *in vivo* and *in vitro* urmylation reactions revealed the covalent attachment of the di-glycine motif via an isopeptide bond to the ɛ-amino-group on a wide variety of substrate lysine residues (Fig. 3c, Supplementary Fig. 5, and Supplementary Table 3A and B). In total, 29 distinct substrate modifications were identified by the *in vitro* approach, while 25 modifications were observed following the *in vivo* Urm1 overexpression (Supplementary Table 3A and B). Interestingly, several of these components tallied with the proteins predicted to be involved in urmylation pathways by a bioinformatic analysis of the genomic context surrounding the *Urm1* gene (see Supplementary Note and Supplementary Fig. 3). For example, *in vitro* modifications were identified on the proteasome β-subunit (Saci0662), and fructose 1,6-bisphosphatase (FBP), while conjugates on a proteasome assembly chaperone (PAC2; Saci0658), PAN (Saci0656), an ATPase RNAaseL inhibitor (RLI), and a HerA/FtsK/TrwB superfamily protein^49^,^50^ (Saci0667) were observed in the *in vivo* analysis (Supplementary Table 3A and B). Furthermore, *in vivo* and *in vitro* modifications were also detected on several ribosomal subunits, and also on a number of tRNA synthases (Supplementary Table 3A and B). Conjugates were also detected on a number of metabolic enzymes, in addition to FBP, including phosphoglycerate kinase (PGK), phosphoenolpyruvate (PEP) synthase, aldehyde dehydrogenase, alcohol dehydrogenase, and acetyl-CoA synthetase (Supplementary Table 3A and B). Thus, it appears that urmylation may also be involved in the regulation of key metabolic pathways.

The identification of Urm1 modifications on the core 20S proteasome, the PAN proteasome regulatory ATPase and the PAC2 proteasome assembly chaperone provided further evidence to suggest a link between urmylation and the proteasome degradation pathway. Modifications were also observed on the Urm1 protein itself in both the *in vitro* and *in vivo* analyses (Supplementary Table 3A and B). These findings were suggestive that multiple lysines on Urm1 can be modified, and polymeric chains could be formed, consistent with the SAMP linkages that have been reported previously^27^,^28^,^29^. Since ubiquitin chains have well-established roles in proteasome-mediated degradation, it remains possible that Urm1/SAMP1 chains may also be used for targeting to the proteasome in the archaea. However, it should be noted that the lysine residues modified in this study do not appear to be conserved across archaeal species (Supplementary Table 3A and B; Supplementary Fig. 1).

### *In vitro* assembly of the *S. acidocaldarius* 20S proteasome

In order to explore the possible physical and functional association between Urm1 conjugates and archaeal proteasome apparatus we biochemically reconstituted the 20S core proteasome from *S. acidocaldarius*. Archaeal 20S proteasomes, like the homologous eukaryotic apparatus, are formed from four stacks of heptameric rings, creating an elongated hollow chamber. Proteasomes from the archaea *Thermoplasma acidophilum*^51^,^52^, *Archaeoglobus fulgidus*^53^ and *Methanosarcina thermophila*^54^ had previously been reconstituted by recombinant expression in *E. coli*. In these cases the proteasome is formed from only two 20S core proteasome proteins; an α-subunit, which produces the self-assembling homo-heptameric α-ring outer rings, and a catalytic β-subunit that constitutes the innermost rings. In contrast, the *S. acidocaldarius* genome encodes two catalytic β-subunit homologues (Saci0662 and Saci0909), in addition to a single α-subunit homologue (Saci0613).

**Figure 4:**
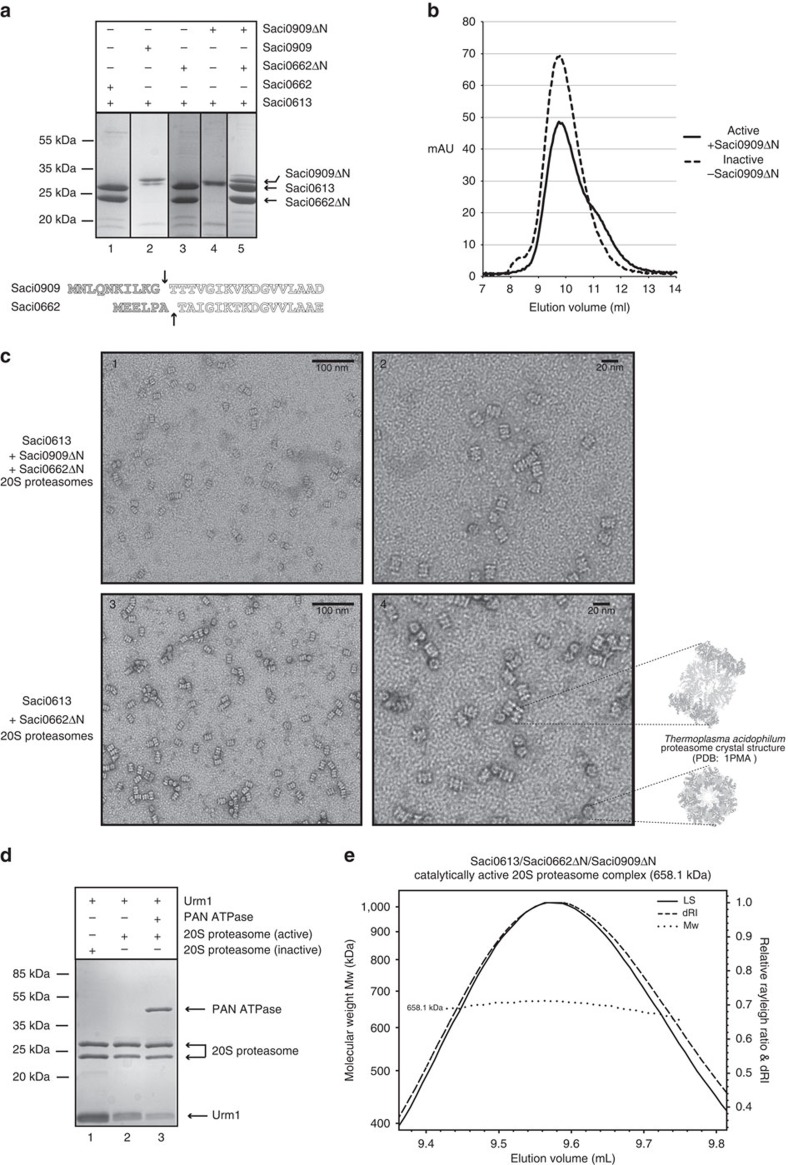
*In vitro* reconstitution of the *S. acidocaldarius* 20S proteasome. (**a**) Ni-NTA agarose IMAC chromatography purification step following coexpression of the untagged Saci0613 α-subunit with the Saci0662 (His-tagged) and Saci0909 β-subunits. Lane 1: Saci0613 plus Saci0662; lane 2: Saci0613 plus Saci0909, lane 3: Saci0613 plus Saci0662ΔN, lane 4: Saci0613 plus Saci0909ΔN; lane 5: Saci0613 plus Saci0662ΔN and Saci0909ΔN. The truncated regions in Saci0662ΔN and Saci0909ΔN, respectively, are indicated by vertical arrows in the schematic below. (**b**) Size exclusion chromatography (SEC) analysis of the Saci0613/Saci0662ΔN/Saci0909ΔN catalytically active, and Saci0613/Saci0662ΔN inactive 20S proteasome complex. Chromatography UV traces are displayed for both elution profiles. Both peaks are consistent with a complex of ≈660 kDa. (**c**) Transmission electron micrographs (TEM) of the Saci0613/Saci0662ΔN/Saci0909ΔN 20S complex (1 and 2), and the Saci0613/Saci0662ΔN inactive 20S complex (3 and 4), negatively stained with uranyl acetate. The left hand panels are at 50 000X magnification and the right hand panels at × 100 000 magnification; 100 or 20 nm scale bars are shown, respectively. (**d**) Free Urm1 is processed by the active 20S proteasome complex, and degradation is stimulated by the addition of the PAN regulatory ATPase. 30 μg of Urm1 protein was incubated with 20 μg of the proteasome, with or without 20 μg of the PAN ATPase complex for 1 h at 70 °C; all reactions contained 5 mM ATP. Lane 1: Urm1 control with the inactive proteasome; lane 2: Urm1 degradation by the active proteasome; lane 3: Urm1 degradation by the active proteasome stimulated by the PAN ATPase. (**e**) SEC-MALS analysis of the Saci0613/Saci0662ΔN/Saci0909ΔN catalytically active 20S proteasome. The complex has a fitted molecular weight of 658.1 kDa (±0.166%), consistent with a 28-subunit cylindrical proteasome assembly, with a polydispersity of 1.000 (±0.234%). Differential refractive index (dRI) and light scattering (LS) are plotted in conjunction with molecular weight (M_w_).

We performed heterologous co-expression trials in *E. coli* with the Saci0613 α–subunit, in combination with either the Saci0662 or Saci0909 β-subunits. In both cases the β-subunit was C-terminally His-tagged, while the α-subunit was native and untagged. We reasoned that if the 20S core subunits were expressed with the correct stoichiometry in *E. coli* then the entire 28-subunit complex should purify over immobilized metal ion affinity chromatography (IMAC). Indeed, a seemingly stoichiometric complex of both α and β components was purified following co-expression of Saci0662 with the α-subunit. By contrast, the α-subunit did not co-elute with the tagged Saci0909 β-subunit following co-expression and purification over the Ni-NTA agarose column (Fig. 4a). Although the Saci0613 α/0662 β proteasome initially appeared stoichiometric, the complex was not stable over size exclusion chromatography (Supplementary Fig. 6). We noted that during 20S assembly in both eukaryotic and prokaryotic proteasomes, an N-terminal pro-peptide is commonly cleaved from the β-subunits to expose a catalytic threonine (T1)^55^. Equivalent threonine residues were identified in both of the *S. acidocaldarius* β-subunits (Fig. 4a), and the β-subunit expression constructs were therefore redesigned to exclude the preceding N-terminal pro-peptides. The truncated β-subunits were then co-expressed with the α-subunit. As observed with the full-length Saci0909β protein, the truncated version of this β-subunit failed to form a stable complex with the α-subunit (Fig. 4a). In contrast, truncation of the 0662β–subunit stabilised the 20S assembly, and this material remained stoichiometric during size-exclusion chromatography (Fig. 4b), eluting at a volume consistent with a 28-subunit complex of 660 kDa. Furthermore, examination of the purified complex by transmission electron microscopy (TEM) revealed a cylindrical arrangement of four stacked heptameric rings, reminiscent of the overall shape of the previously determined *T. acidophilum* 20S core proteasome crystal structure^51^ (Fig. 4c).

Although co-expression of the 0613 α-subunit and the truncated 0662 β-subunit resulted in a macromolecular assembly that resembled the structures of the active *T. acidophilum*, *M. thermophilus* and *H. volcanii* proteasomes^51^,^54^,^55^, this material showed no catalytic activity (Fig. 4d). Consequently, both the N-terminally truncated 0662 and 0909β-subunits were simultaneously co-expressed with the α-subunit. This method resulted in formation of another stable complex, again consistent with a 28-subunit complex, as verified by TEM and SEC–MALS (size-exclusion chromatography–multi-angle laser light scattering) (Fig. 4c,e). However, the complex was now composed of all three subunits, with the Saci0909β subunit incorporating into the β-subunit ring at a lower ratio than the Saci0662 subunit (Fig. 4a). Critically, the inclusion of the Saci0909 β-subunit into the 20S core conferred catalytic activity to the complex, as demonstrated by the direct degradation of the Urm1 modifier by the core 20S proteasome (Fig. 4d).

### Urm1 conjugates engage with the active 20S proteasome

**Figure 5:**
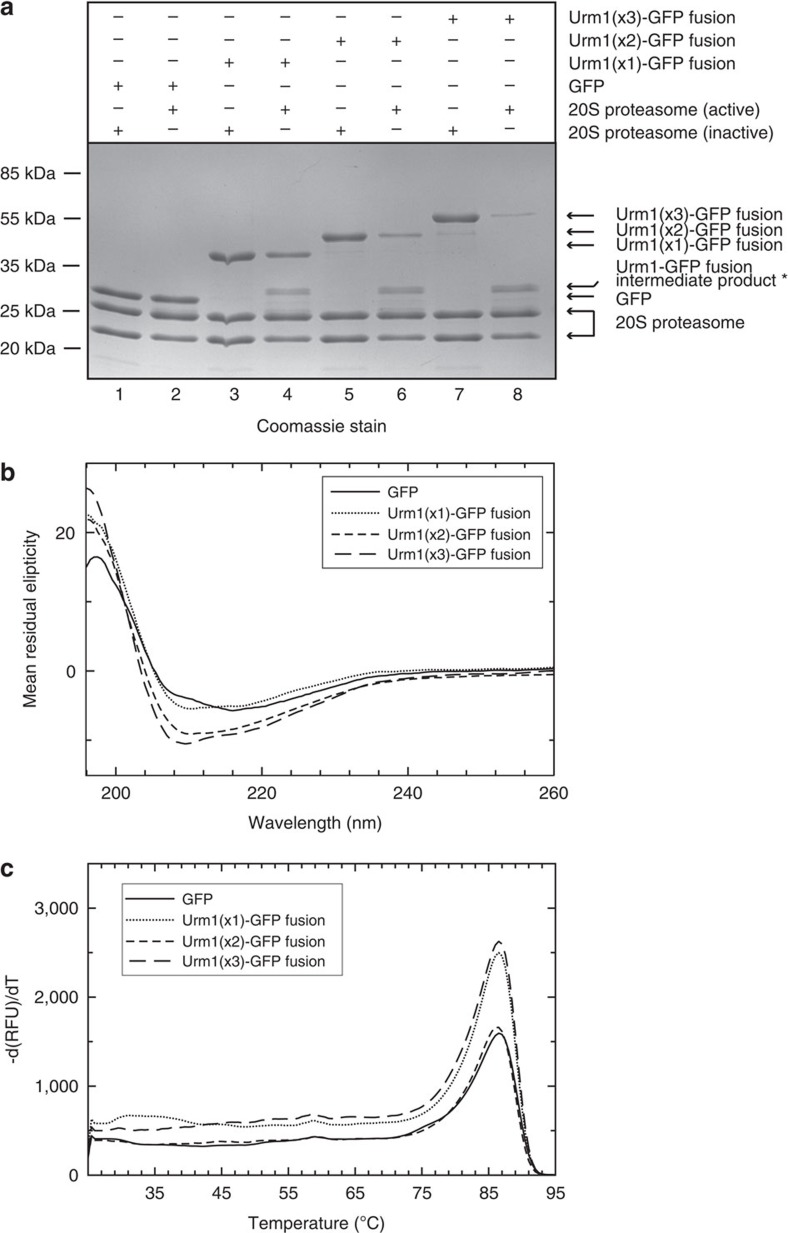
N-terminal fusion of Urm1 to a GFP substrate results in targeting to the 20S proteasome. (**a**) N-terminal fusion of Urm1 (or a tandem chain of two, or three, Urm1 subunits) to a thermally stable GFP^56^ substrate results in processing by the 20S proteasome. 10 μg of substrate was incubated with 20 μg of the proteasome complex for 1 h at 70 °C. Reactions using the Saci0613/Saci0662ΔN/Saci0909ΔN active complex are shown in lanes 2 (GFP), 4 (1XUrm1:GFP), 6 (2XUrm1:GFP) and 8 (3XUrm1:GFP); lanes 1, 3, 5 and 7: negative controls for each of the GFP substrates, respectively, using the inactive Saci0613/Saci0662ΔN complex. The asterisk denotes an intermediate product in the processing reaction (mass spectrometry analysis of this product is provided in Supplementary Fig. 8). (**b**) Circular dichroism spectra of GFP and the Urm1:GFP fusion proteins, revealing that Urm1 fusion does not affect GFP folding. The CD spectrum for GFP alone shows negative and positive Cotton effects at about 216 and 198 nm, respectively. This indicates a β-strand dominated structure as expected. Addition of Urm1 domains to GFP resulted the appearance of another minima at about 208 nm which is due to the parallel component (relative to α-helix axis) of the *π* to π* transition in Urm1 α-helices. In addition, there is a shift in the positive cotton effect maximum from 198 nm towards shorter wavelength, which is probably due to the perpendicular component of the π to π* transition in Urm1 α-helices. The absence of a clear negative band at 222 nm due to the *π* to π* transition, which is a signature of α-helices, is most likely an indication of the relative small amount of helices in Urm1. (**c**) Thermal denaturation assay analyses of GFP and the Urm1:GFP fusion proteins, measuring the innate fluorescence of (superfolder) GFP^56^ (490-nm excitation with emission at 510 nm). The first derivative plot is displayed (change in relative fluorescence units (RFU) with time [dRFU/dT]). The maximum dRFU/dT occurs at 85 °C in all samples, indicating that addition of Urm1 to the N terminus of GFP does not affect the folding or stability of the GFP substrate.

Our bioinformatic and mass-spectrometry analyses were suggestive of a link between Urm1 and the proteasome, consistent with previous studies^27^,^33^,^44^. However, no categorical demonstration of a physical interaction between an archaeal Ubl and the archaeal 20S proteasome has been shown thus far. Having reconstituted the active *S. acidocaldarius* 20S core proteasome *in vitro*, we proceeded to perform a series of biochemical assays to explore the potential association between the proteasome and the urmylation pathway. Unexpectedly, we found that the active 20S core proteasome complex, with the incorporated catalytic Saci909 β-subunit, was competent to process the Urm1 protein directly (Fig. 4d). By contrast, we found that a thermally stable (superfolder) green fluorescent protein (GFP)^56^ control protein was not processed under the same conditions (Fig. 5a), suggesting that the Urm1 protein is recognized specifically by the cylindrical protease complex. Furthermore, we also expressed the Saci0656 PAN protein, a homohexameric ATPase homologous to the Rpt hetero-hexameric ring of the eukaryotic proteasome, which interacts with the α-ring of the core proteasome^57^. SEC-MALS analysis confirmed the expected hexameric status of this complex (Supplementary Fig. 7). We observed that inclusion of PAN in the assay resulted in a clear stimulation of the Urm1 degradation (Fig. 4d).

After demonstrating that the Urm1 itself is processed by the archaeal proteasome, we next investigated if fusion of Urm1 to a target protein would stimulate degradation of this substrate. N-terminal fusion of Urm1 to the superfolder GFP resulted in substrate association with the 20S proteasome and clear degradation of the Urm1 portion of the substrate; crucially the GFP protein was not degraded in the absence of the fused Urm1 tag (Fig. 5a). Furthermore, addition of two or three Urm1 chains, linked in tandem to the GFP substrate, resulted in enhanced substrate processing (Fig. 5a). Circular dicroism (CD) spectroscopy and thermal shift analyses verified that the GFP fusion proteins remained correctly folded, and were not destabilised upon fusion with Urm1 (Fig. 5b,c). In each reaction, an intermediate product was generated, slightly larger than the untagged GFP substrate (Fig. 5a). Mass spectrometry analysis confirmed that this product consisted of a short C-terminal Urm1 peptide attached to the full length GFP protein (Supplementary Fig. 8). This result suggested that the Urm1 tag of these fusion proteins engaged and entered the 20S core, but the processing reaction paused, or stalled, as the fused GFP substrate accessed the proteasome (Fig. 5a, Supplementary Fig. 8).

**Figure 6:**
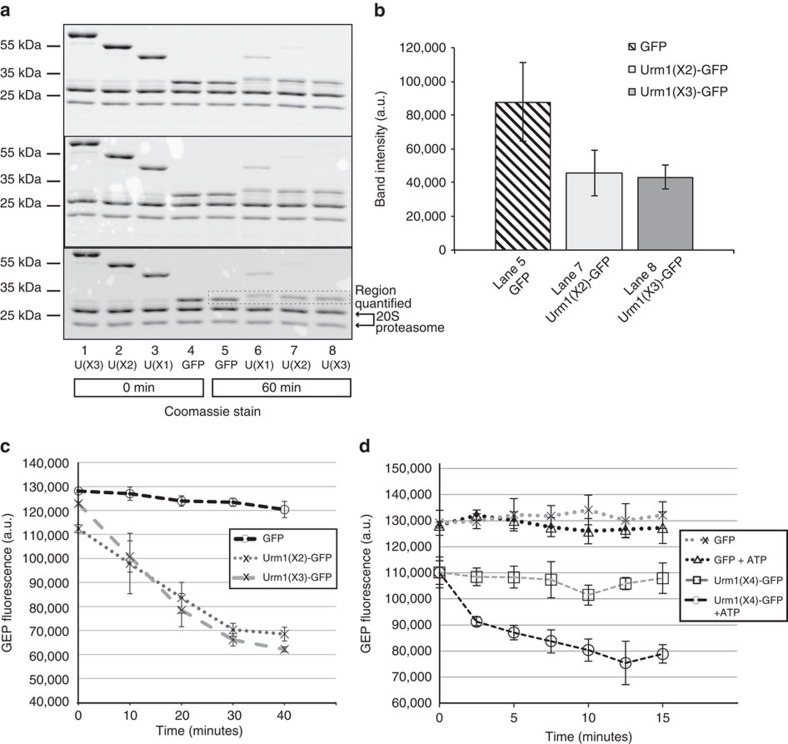
Quantification of the Urm1-GFP fusion substrate processing by the core 20S proteasome, and unfolding by the PAN ATPase. (**a**) 20S core processing of the superfolder GFP protein and the 1XUrm1:GFP, 2XUrm1:GFP and 3XUrm1:GFP N-terminal fusion substrates in triplicate and visualized by SDS-PAGE and Coomassie staining. Equimolar quantities (6 μM) of the substrates were incubated with 10 μg Saci0613/Saci0662ΔN/Saci0909ΔN active 20S complex at 69 °C. Lanes 1–4 display the reactions before heating, while lanes 5–8 represent the reactions upon termination after 75 min at 69 °C. (**b**) Quantification of the GFP-sized intermediates (boxed region) after resolution by SDS-PAGE. Gels were scanned using the ‘Coomassie Brilliant Blue Digitization' mode on a Typhoon Imaging system (GE Healthcare). The histograms display the mean of three independent repeats and the error bars one s.d. The data were quantified using the ImageQuant software (GE Healthcare) and plotted in Microsoft Excel. (**c**) Verification of the direct degradation of the Urm1-GFP fusion substrates by the 20S core proteasome (at 69 °C), demonstrated by the reduction in the GFP fluorescence. (**d**) ATP-dependent unfolding of a (4X) Urm1:GFP fusion protein by the PAN ATPase (at 60 °C) reflected by the decrease in the GFP fluorescence. Untagged GFP is not unfolded by PAN, even in the presence of ATP. In (**c**,**d**) the data points represent the mean of three independent repeats and the error bars one s.d. Data were plotted in Microsoft Excel. The GFP fluorescence was detected at 485 nm excitation and 520 nm emission wavelengths, respectively, using a PheraStar (BMG LABTECH) plate reader.

Although it was apparent that the Urm1 region of the GFP fusion proteins were efficiently processed by the active 20S core, it was less obvious if a proportion of the fused GFP also entered the 20S proteasome. We therefore performed the degradation assays in triplicate using equimolar ratios of the GFP substrates, and then quantified the Coomassie-stained intermediate products, and compared these with the equivalent band in the untagged GFP control. Unexpectedly, this quantification suggested that a proportion of the tagged GFP substrates were also entering and being processed by the active 20S core (Fig. 6a,b). This finding was also confirmed by directly measuring the GFP degradation in real-time by following the change in GFP fluorescence throughout the reaction (Fig. 6). It should be noted that in other systems the stably folded GFP proteins are resistant to processing by core proteasomes in the absence of an ATP-driven unfoldase^58^,^59^. To our knowledge, this is the first time that the *S. acidocaldarius* 20S proteasome has been examined to date, and whereas previous studies using other archaeal 20S cores were performed at 45 °C (ref. 58), our assays were performed at the more physiological temperature of 70 °C. A control reaction using AMP-PNP (a non-hydrolysable analogue of ATP) ensured that the observed GFP processing was not mediated by a contaminating *E. coli* ATPase (Supplementary Fig. 9).

We also examined the ability of the PAN regulatory ATPase to recognize and unfold the Urm1-GFP fusion proteins, measuring changes in the GFP fluorescence as a readout for substrate unfolding. In contrast to the untagged GFP control, which remained stable even when ATP was included in the reactions, the Urm1-GFP fusion protein was unfolded by the PAN protein in an ATP-dependent manner (Fig. 6d). Taken together, our results provide the first biochemical evidence that Urm1/SAMP tagged substrates are targeted to the archaeal 20S proteasome for destruction, by either direct interaction with the 20S core or via the PAN regulatory ATPase.

**Figure 7:**
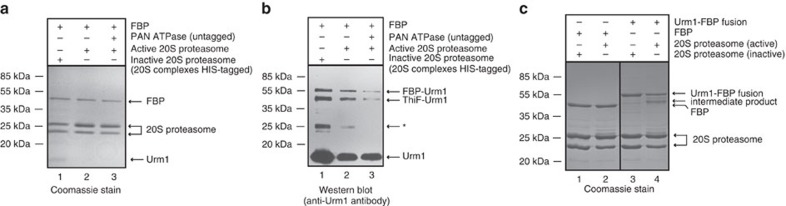
FPB substrates are directed to the 20S proteasome as a result of either Urm1 N-terminal fusion, or by modification on internal lysines using the ELSA/Uba4p/ThiF E1 enzyme. (**a**,**b**) *In vitro* urmylation of FBP and subsequent proteasomal processing by the core 20S and PAN-20S complexes. 100 μg FBP, 30 μg N-terminally His-tagged Urm1 protein and 7.5 μg ELSA/Uba4p/ThiF enzyme, plus ATP at 3 mM were incubated together at 70 °C for 1 h in a 200 μl reaction. 50 μl of the urmylated reaction was then incubated at 70 °C for 1 h with 20 μg 20S proteasome, with or without the addition of 20 μg PAN ATPase, and a further 12.5 mM ATP. Products were purified by Ni-NTA pulldown before resolution by SDS-PAGE. (**b**) Urmylated products were visualized by Western blot probed with an anti-Urm1 antibody. Lane 1: Urmylated FBP reaction incubated with the inactive proteasome (negative control); lane 2: urmylated FBP reaction with processing by the active 20S proteasome; lane 3: stimulation of the processing reaction by the active proteasome following addition of the PAN ATPase. Arrows indicate the urmylated FBP product and also an urmylated ELSA/Uba4p/ThiF band. The band visible at 25 kDa (indicated by an asterisk) is an urmylated truncation product of the ELSA/Uba4p/ThiF protein. Panel (**a**) is a duplicate Coomassie-stained control gel of (**b**) demonstrating that the non-urmylated FBP substrate is not processed. (**c**) N-terminal fusion of a single Urm1 moiety to an FBP substrate results in targeting to the 20S proteasome. 10 μg of substrate was incubated with 20 μg of the proteasome complex for 1 h at 70 °C. Reactions using the active proteasome complex are displayed in lanes 2 (FBP) and 4 (Urm1:FBP). Lanes 1 and 3: negative controls for FBP and Urm1:FBP fusion, respectively, using the inactive proteasome.

Although ubiquitin may sometimes become conjugated to the N terminus of substrates in eukaryotes^60^, in addition to the well-characterised modifications on internal lysine residues, it is not yet known if Urm1/SAMP proteins can attach to the N terminus of target proteins *in vivo*. We therefore performed an *in vitro* urmylation of the FBP protein, to generate intermediates conjugated to the lysine residues on the substrate and then presented this modified material to the 20S proteasomes. While the unmodified FBP protein was not processed by the proteasome (Fig. 7a), the urmylated FBP material associated with the 20S core, resulting in the processing of at least the Urm1 portion of the fused substrate (Fig. 7b); notably this processing was stimulated in the presence of PAN regulatory ATPase (Fig. 7b). Furthermore, an N-terminal fusion of the Urm1-tag to FBP also resulted in targeting of the substrate to the 20S core proteasome (Fig. 7c). Again, an intermediate product accumulated indicative of the reaction stalling at the FBP region of the substrate. It therefore appeared that, in the absence of a regulatory unfoldase, the Urm1-FBP fusion substrates stalled at the 20S proteasome core after efficient degradation of the Urm1 tag, as was observed with the Urm1-GFP proteins.

## Discussion

In eukaryotic cells, the ubiquitin-mediated proteasomal degradation pathway plays critical roles in controlling diverse molecular processes through the regulation of protein turnover^1^,^2^. Similarly, analogous bacterial systems, using the Pup modifier, target a variety of substrates for destruction at the archaeal/eukaryotic-like 20S proteasomes, found exclusively in the *Nitrospirae* and *Actinobacteria* families^35^,^36^,^37^,^38^,^39^. However, Pup is a functional analogue of ubiquitin^43^, rather than a homologue and seems to have evolved independently from the Ub/Ubl family. Since the discovery of the SAMP family of proteins in the archaea^27^, these ancestral Ubls have been implicated in proteasomal processing pathways equivalent to the eukaryotic ubiquitin-proteasome system. However, despite this premise, direct evidence of a physical association between SAMPs and the archaeal proteasome has remained elusive to date.

Our current study has identified a SAMP homologue in *S. acidocaldarius*, which displays remarkable structural and amino-acid sequence similarity to the eukaryotic ubiquitin-like modifier, Urm1. We have revealed that this protein interacts directly with the archaeal 20S core proteasome, and is degraded by this protease complex. It should be noted that most natively folded proteins, and even aggregated polypeptides, are generally not processed by 20S proteasomes^61^. Therefore, the Urm1 degradation appears to result from a specific recognition of the modifier by the protease complex. Furthermore, the inclusion of the *S. acidocaldarius* PAN regulatory ATPase in this reaction stimulates free Urm1 degradation (Fig. 4d). Crucially we find that covalent attachment of Urm1 to a substrate, either by N-terminal fusion, or by *in vitro* urmylation on lysine residues of the target, results in recognition of the modified protein directly by the core 20S proteasome and also by the PAN regulatory ATPase. Regardless of the attachment site, the Urm1 portion of the conjugate, or fused substrate, was processed directly by the 20S core proteasome. However, the larger globular GFP and FBP regions of these substrates accumulated as intermediates during the processing reactions (Figs 5, 6, 7). It therefore seems most likely that regulatory unfoldases, such as PAN, are required for the efficient degradation of stably folded globular substrates in *S. acidocaldarius* cells, consistent with previously described energy-dependent protease complexes in other organisms^59^. Indeed, we observed that the PAN ATPase specifically recognized and unfolded an Urm1-GFP fusion in an ATP-dependent manner (Fig. 6d). It was also clear that the substrates in our assays lacking an Urm1 tag remained refractory to proteasomal processing. Thus, it appears that the covalent conjugation of small modifier proteins to mark substrates for proteasomal destruction is a feature common to eukaryotes and archaea, and also in the 20S proteasome-bearing bacterial species.

**Figure 8:**
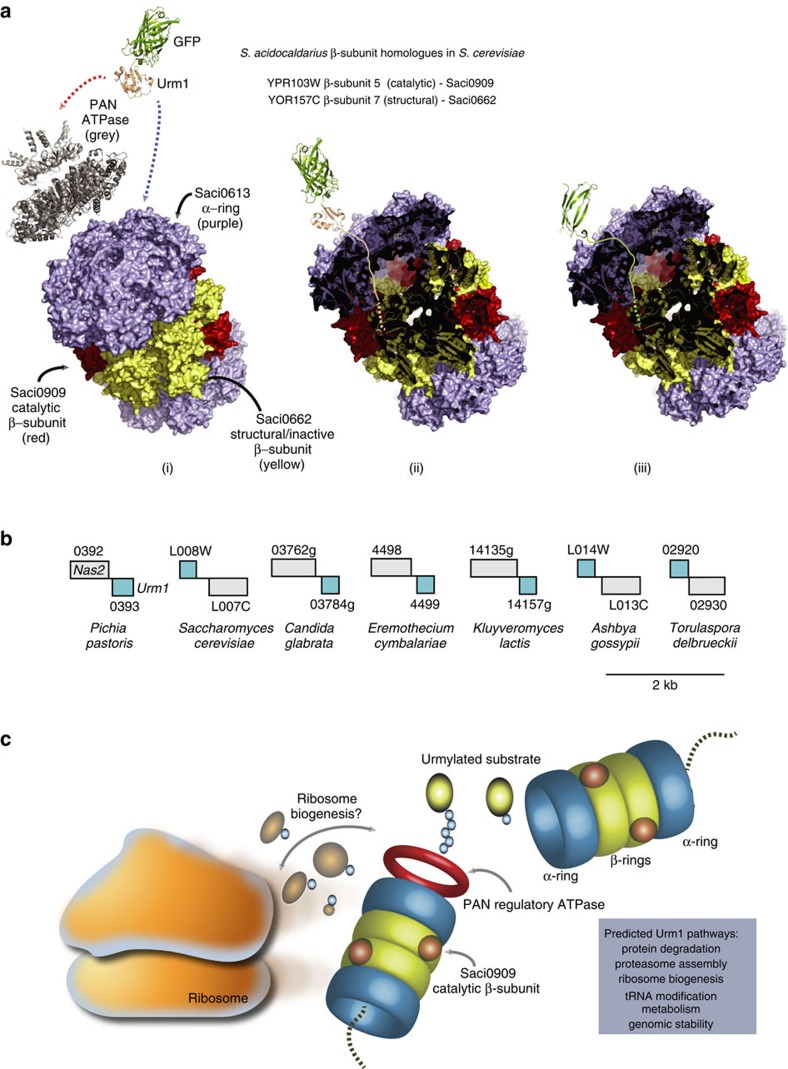
Proposed models for the archaeal Urm1-proteasome system. (**a**) Covalent attachment of Urm1 to substrates results in association with either the core 20S proteasome (blue dashed arrow) or the PAN regulatory ATPase (red dashed arrow) *in vitro*. The Urm1 tag leads the entry of the fused GFP substrate (green) into either complex. (i) For illustrative purposes the *T. acidophilum* 20S proteasome crystal structure (PDB:1PMA) is displayed. The 20S outer α-rings are coloured purple (equivalent to Saci0613), while the inner β-rings are shown in yellow (representing Saci0662). Three of the α-subunits are coloured red to represent the Saci0909 active catalytic subunit (Inset; yeast homologues of the *S.acidocaldarius* β-subunits identified by BLAST, see Supplementary Table 4). The *Methanocaldococcus jannaschii* PAN complex (grey) is also displayed in a view perpendicular to the central channel (upper domains; the PAN N-terminal hexameric ring (PDB:3H43 (ref. 69)); *below;* the monomeric PAN nucleotidase domain structure (PDB:3H4M) modelled onto the coordinates of hexameric HslU (PDB:1DO0)^69^); (ii) cutaway view of the proteasome cylinder to demonstrate processing of the Urm1 tag of the Urm1:GFP fusion proteins by the core 20S, in a manner reminiscent of the bacterial Pup-proteasome system; (iii) this study suggests that some stably-folded Urm1-tagged substrates may enter the 20S core even in the absence of an unfoldase at physiological temperatures. (**b**) Genomic context associations between eukaryotic *Urm1* gene (blue) and the *Nas2* proteasome assembly chaperone gene (light grey) in a wide variety of yeast species, suggestive of a putative role for Urm1 in assembly of the eukaryotic proteasome. ORFs positioned above the midline are transcribed left to right, and those below the line transcribed right to left. (**c**) Schematic model of the archaeal Urm1-proteasome system, and the hypothetical role in the assembly and maintenance of the archaeal ribosome. Urm1 modifications have been detected on several ribosome subunits in this study, while ubiquitin is proposed to play roles in eukaryotic ribosome maintenance^70^. Our study also implicates the archaeal Urm1 protein in proteasome assembly pathways, tRNA modification, metabolism, and the maintenance of genomic stability by DNA repair (see Supplementary Note, Supplementary Fig. 3 and Supplementary Tables 3 and 5 for further details).

Unlike the eukaryotic ubiquitin associated degradation system, where the modifier is cleaved from the target and recycled at the proteasome^62^, the archaeal Urm1 protein, is destroyed by the *S. acidocaldarius* core 20S proteasome *in vitro.* Thus, the modifier apparently leads the process, guiding covalently conjugated substrates into the proteasomal cylinder (Fig. 8a). Our quantification of the 20S proteasomal processing assays suggested that a proportion of the substrates entered the proteasomal 20S core, even in the absence of a regulatory ATPase. This contrasts with previous studies of other archaeal proteasome complexes, and may reflect the fact that our assays are performed at the physiological temperature of 70 °C, rather than the 45 °C temperature used in earlier investigations^58^. The mechanism of the Urm1 modifier leading the substrate destruction is highly reminiscent of the bacterial Pupylation pathway, in which the N terminus of the Pup modifier is first threaded into the core of the protease, via the Mpa regulatory ATPase, leading to the degradation of the modifier and subsequently the covalently attached substrate^42^. Interestingly, deconjugation of Pup from modified targets, by the Dop protein, has been reported previously^63^,^64^, while SAMPylated proteins in *H. volcanii* can be de-SAMPylated by JAB1/MPN/MOV34 metalloenzymes^65^.

Our demonstration of the physical and functional association between the Urm1 protein and the archaeal proteasome necessitated the reconstitution of the *S. acidocaldarius* 20S assembly *in vitro* for the first time. This revealed that the central catalytic β-rings of this complex are composed of two different subunits. In contrast, the β-rings of previously purified archaeal 20S proteasomes from *H. volcanii*, *T. acidophilum* and *M. thermophilum* are homo-heptameric^51^,^54^,^55^. In *S. acidocaldarius*, the first of the two β-subunits is seemingly catalytically inert, and instead appears essential for interaction with the outer α-rings to form the 20S cylinder. The second β-subunit then appears to intersperse within the β-ring, at a low stoichiometry relative to the other subunits, to provide catalytic activity (Figs 4a,d and 8a). Parallels can been made with the eukaryotic 20S complex in which only three of the seven β-subunits confer catalytic activity^39^,^52^. Indeed, BLAST searches reveal that *S. acidocaldarius* catalytic β-subunit is most similar the *S. cerevisiae* β5 catalytic subunit, whereas the other structural β-subunit is more homologous to the non-catalytic β7 in yeasts (Fig. 8a and Supplementary Table 4).

The structural, bioinformatic and functional data presented here add further support to the theory that the archaeal and eukaryotic Urm1 proteins are closely related, and have evolved from a shared common ancestor^10^. However, in eukaryotes, a direct association between the Urm1 modifier and the proteasome complex has not been identified to date. It is clear that the proteasome-mediated degradation pathway has been appropriated by the more sophisticated and adaptable ubiquitylation system in complex eukaryotic organisms^24^. However, has the ancient association of Urm1 with the proteasome that we still observe in the archaeal domain of life been entirely usurped by the ubiquitin system in the eukaryotes? In a final bioinformatic observation, we note that in a variety of yeast species the genes encoding for Urm1 and the Nas2 proteasome assembly chaperone^66^ are immediately juxtaposed (Fig. 8b). This genomic linkage is suggestive of an involvement of Urm1 in the eukaryotic proteasome assembly process. Interestingly, there is some precedent for Urm1 playing a role in the assembly of the archaeal proteasome, as we detected *in vivo* and *in vitro* Urm1 modifications on the *S. acidocaldarius* PAC2 proteasome assembly chaperone (Supplementary Table 3A and B). It is tempting to speculate that in the ancestral archaeal system, Urm1 may play roles in both proteasomal assembly and substrate destruction pathways. Similarly, it remains plausible that in eukaryotic organisms Urm1-conjugated proteins could still interact with proteasome, at least during assembly of the protease complex.

The Urm1/SAMP mediated proteasome degradation pathway identified by this study is representative of a streamlined and presumably ancestral version of the eukaryotic ubiquitin-proteasome degradation system. It remains to be determined if Urm1 marked substrates are recognized directly by the core 20S proteasome *in vivo*, in accordance with our observations using the *in vitro* reconstituted *S. acidocaldarius* system. This may represent an additional processing mechanism operating alongside the established degradation routes that utilize the regulatory ATP-dependent unfoldases, as reported in other systems^59^. Similarly, further studies should also reveal if the conjugation of a single Urm1 protein is sufficient to target substrates to the proteasome *in vivo*, or whether multiple Urm1 moieties are required for efficient processing. It will also be important to investigate if Urm1 is deconjugated and recycled at the proteasome before substrate delivery, or destroyed *in vivo* along with the target, as we observe with the reconstituted 20S proteasome. Our findings to date are also suggestive that the *S. acidocaldarius* Urm1 modifier may regulate a variety of diverse cellular processes and pathways (see Supplementary Note and Fig. 8c). Intriguingly, many equivalent biological functions in eukaryotic organisms are also regulated by the Urm1 and Ub modifiers. Thus, the adoption of simple and biochemically tractable archaeal models to investigate ubiquitin-like pathways should provide us with valuable future insights into these key conserved mechanisms.

## Methods

### Protein purification

Open reading frames (ORFs) were amplified by PCR from *S. acidocaldarius* DSM639 genomic DNA, cloned into either pET30a, pET33b, pET28a, pCDF-DUET or pET-DUET vectors (Novagen), and expressed in *E. coli* Rosetta (DE3) pLysS cells (Novagen). Cell lysates were heat-clarified at 70 °C for 20 min. His-tagged soluble proteins were subsequently purified by Ni-NTA IMAC and size exclusion chromatography. Full experimental details of the cloning strategy and purification procedure for each protein (including the untagged Urm1 and ELSA/Uba4p/ThiF proteins) are provided in the Supplementary Methods and Supplementary Table 6.

### Crystallization and X-ray structure determination

The crystal structure of *S. solfataricus* Urm1 was determined by molecular replacement of a native crystal data set, leading to a complete structure refined to a resolution of 2.2 Å. Full details of the structure determination and refinement are given in the Supplementary Methods.

### *In vitro* urmylation assays

Target protein (75 μg), 30 μg N-terminally His-tagged Urm1 protein and 15-μg ELSA/Uba4p/ThiF enzyme were incubated together at 70 °C for 1 h in either the presence or absence of 2.5-mM ATP in 200-μl urmylation reaction buffer (20 mM Tris acetate (pH 8.0), 100 mM NaCl, 5 mM MnCl_2_, 10 mM MgOAc, 50 mM KOAc, 1 mM Zn(OAc)_2_, 5% glycerol and 0.02% β-mercaptoethanol). Urmylated products were then retrieved by Ni-NTA pulldown, washed in TBST (10 mM Tris (pH 8.0), 150 mM NaCl, 0.1% Tween20) supplemented with 15 mM imidazole, and the products eluted by boiling in 2 laemmli protein loading buffer. Urm1 conjugates were then separated by SDS-PAGE, and identified by western blot.

### *In vitro* urmylation of *S. acidocaldarius* cell extract for MS analyses

*S. acidocaldarius* cell-free extracts were prepared by collecting 1 l of cells at OD_600nm_ at 0.6, washed in 1X TBS (10 mM Tris (pH 8.0), 150 mM NaCl), and resuspended in 5 ml 1X TBS, 0.1% Triton-X-100, 0.1% β-mercaptoethanol and 1X EDTA-free protease inhibitors (Roche). Cells were disrupted by sonication and the lysate clarified by centrifugation. 250 μl of the cell-free extract was added to 500-μl urmylation reaction buffer, supplemented with 375 μg N-terminally His-tagged Urm1 protein, 375 μg ELSA/Uba4p/ThiF enzyme and 10-mM ATP, and incubated for 1.5 h at 70 °C. Urmylated products were purified by Ni-NTA pulldown, separated by SDS-PAGE, and analysed by GeLC-MS/MS.

### *In vivo* overexpression of Urm1 for MS analyses

N-terminally hexa-His-tagged Saci0669 (Urm1) was overexpressed in *S. acidocaldarius* strain MW001 with the vector pCMalLacS^67^, following induction with 0.4% maltose. Urm1 conjugates were purified from cell lysate by IMAC on Ni-NTA agarose. Full experimental details are described in the Supplementary Methods.

### GeLC-mass spectrometry and MS data analysis

All GeLC-MS/MS experiments were performed using a nanoAcquity UPLC (Waters Corp., Milford, MA) system and an LTQ Orbitrap Velos hybrid ion trap mass spectrometer (Thermo Scientific, Waltham, MA). Full experimental details including sample preparation and data analysis are provided in the Supplementary Methods.

### Size exclusion chromatography–multi-angle laser light scattering

Samples analysed by SEC-MALS (100 μl protein complex at 2 mg.ml^−1^) were passed over a Superdex 200 10/300 Increase GL column (GE Healthcare), in 20 mM Tris (pH 8.0), 300 mM NaCl. The column output was fed into a DAWN HELEOS II MALS detector with a laser source at 664 nm, and eight fixed angle detectors (Wyatt Technology), followed by an Optilab T-rEX differential refractometer, using a 664 nm LED light source at 25 °C (Wyatt Technology).

### Reconstitution of the *S. acidocaldarius* 20S proteasome

Full length untagged Saci0613 α ORF was coexpressed with N-terminally truncated Saci0662 β, with or without the N-terminally truncated Saci0909 β catalytic subunit in *E. coli* Rosetta (DE3) pLysS cells (Novagen). A 28-subunit (∼660 kDa) complex was purified by heat clarification of the extract, Ni-NTA agarose IMAC, and size exclusion chromatography. Full experimental details of the cloning strategy and purification procedure are provided in the Supplementary Methods.

### Electron microscopy

*S. acidocaldarius* 20S proteasome complexes were visualized with an FEI Philips CM100 transmission electron microscope at the Advanced Imaging Centre, University of Cambridge. 10 μl of the 20S proteasome complex at 0.035 mg ml^−1^ was applied to glow-discharged carbon-coated electron microscopy grids, pre-coated with 10 μl 0.1% poly-L-lysine (Sigma), and negatively stained with 10 μl 2% (w/v) uranyl acetate.

### N-terminal Urm1 fusion protein proteasome processing assays

Ten microgram of substrate (GFP, Urm1:GFP fusion, FBP, Urm1:FBP fusion or native Urm1 protein) was incubated, for 1 h at 70 °C, with 20 μg of either active (including the Saci0909 catalytic β subunit) or inactive (without Saci0909β) 20S proteasome complex in 70-μl proteasome reaction buffer (20 mM Tris acetate (pH 8.0), 100 mM NaCl, 5 mM MnCl_2_, 10 mM MgOAc, 50 mM KOAc, 1 mM Zn(OAc)_2,_ 5% glycerol and 0.02% β-mercaptoethanol). After addition of 2X laemmli protein loading buffer, the products were then separated by SDS-PAGE, and visualized with Coomassie stain. For the quantified assays in Fig. 7 equimolar quantities (6 μM) of the GFP, 1XUrm1:GFP, 2XUrm1:GFP and 3XUrm1:GFP were incubated with 10-μg active 20S complex at 69 °C for 75 min. Gels were scanned using the ‘Coomassie Brilliant Blue Digitization' mode on a Typhoon Imaging system (GE Healthcare), and bands were quantified using the ImageQuant software (GE Healthcare).

### *In vitro* urmylation and proteasome processing assay

100 μg FBP, 30 μg N-terminally His-tagged Urm1 protein and 7.5 μg ELSA/Uba4p/ThiF enzyme were incubated together at 70 °C for 1 h in 200-μl urmylation reaction buffer (20 mM Tris acetate (pH 8.0), 100 mM NaCl, 5 mM MnCl_2_, 10 mM MgOAc, 50 mM KOAc, 1 mM Zn(OAc)_2,_ 5% glycerol, 0.02% β-mercaptoethanol) and supplemented with 3 mM ATP. 50 μl of the urmylated reaction was then incubated, at 70 °C for 1 h, with 20 μg of either catalytically active, or inactive 20S proteasome complex, with or without the addition of 20 μg of the PAN ATPase, in the presence of an additional 12.5 mM ATP, in a final volume of 80 μl. Products were then retrieved by Ni-NTA pulldown, washed in TBST (10 mM Tris (pH 8.0), 150 mM NaCl and 0.1% Tween20) supplemented with 15-mM imidazole. Proteins were eluted by boiling in 2X laemmli protein loading buffer, separated by SDS-PAGE, and visualized with Coomassie stain.

### GFP fluorescence assay measuring 20S degradation and PAN unfolding

For the 20S proteasome core degradation reactions, equimolar quantities (6 μM) of the GFP, 1XUrm1-GFP, 2XUrm1-GFP and 3XUrm1-GFP were incubated with 10 μg active 20S proteasome complex in reaction buffer (20 mM Tris acetate (pH 8.0), 100 mM NaCl, 5 mM MnCl_2_, 10 mM MgOAc, 50 mM KOAc, 1 mM Zn(OAc)_2,_ 5% glycerol and 0.02% β-mercaptoethanol) at 69 °C for 10, 20, 30 or 40 min, respectively. 3 μl sample was then diluted in 107-μl reaction buffer and 100 μl dispensed into the wells of a 96 well ‘Half-area' plate (Corning). For the unfolding assays by the PAN ATPase, 4.65 μM of a 4XUrm1-GFP fusion protein was incubated with 37-μg PAN in a total reaction buffer, with or without the inclusion of 3.5 mM ATP. Reactions were heated at 60 °C for 2.5, 5, 7.5, 10, 12.5 and 15 min, respectively, and then spun to remove any aggregates. 3 μl sample was then diluted in 107 μl reaction buffer and 100 μl dispensed into the wells of a 96 well ‘Half-area' plate (Corning). For both assays the fluorescence signal was detected using a PheraStar (BMG LABTECH) plate reader, at 485-nm excitation and 520-nm emission wavelengths. The GFP sample, equilibrated at room temperature, was used to calibrate the instrument.

### Circular dichroism

CD spectra between 250 and 185 nm were recorded on an AVIV 410 spectropolarimeter (Aviv Biomedical), at 25 **°**C and 0.5 nm steps. The full methodology is provided in the Supplementary Methods.

### Thermal denaturation assay

Melting temperatures were obtained using a 96-well plate format CFX Connect Real Time PCR Detection System (Bio-Rad), equipped with a photodiode detector with FRET channel excitation and emission wavelengths of 490 and 575nm, respectively, suitable for detection of GFP. Each well contained 5-μM protein (as determined by ultraviolet spectroscopy) in 20 mM Tris Cl (pH 8.0), 300 mM NaCl, 5% glycerol and 1 mM DTT buffer in a total volume of 24 μL. Samples were equilibrated at 25 °C for 5 min before increasing the temperature to 95 °C in 0.5 °C increments, taking a fluorescence reading after 30 s settling time with each increment. The fluorescence of the superfolder GFP (Sandia Biotech) fluorophore was followed at 510 nm as a function of temperature. Melting temperatures was obtained as the lowest point of the first derivative plot (dRFU (relative fluorescence units)/dT), as calculated by the Bio-Rad CFX Manager software (Bio-Rad Laboratories, USA).

## Additional information

**Accession codes**: The coordinates and structure factors of the *S. solfataricus* Urm1 crystal structure have been deposited in the Protein Data Bank under accession code 4WWM.

**How to cite this article:** Anjum, R. S. *et al.* Involvement of a eukaryotic-like ubiquitin-related modifier in the proteasome pathway of the archaeon *Sulfolobus acidocaldarius*. *Nat. Commun.* 6:8163 doi: 10.1038/ncomms9163 (2015).

## Supplementary Material

Supplementary Information Supplementary Figures 1-9, Supplementary Tables 1-6, Supplementary Note, Supplementary Method and Supplementary References

## Figures and Tables

**Table 1:** Data collection and refinement statistics.

	***Sso*** **Urm1**
*Data collection*	
** **Space group	P 2_1_ 2_1_ 2_1_
	
*****Cell dimensions*	
** ***a*, *b*, *c* (Å)	31.49 65.19 109.36
** **α, β, γ (°)	90 90 90
** **Wavelength (Å)	1.5418
** **Resolution (Å)	28−2.20 (2.30−2.20)*
** ***R*_sym_ or *R*_merge_	0.0642 (0.3046)
** ***I*/*σI*	27.26 (6.05)
** **Completeness (%)	99.8 (99.3)
** **Redundancy	12.71 (8.55)
** **Wilson B factor	25.60
	
*Refinement*	
** **No. reflections	12.034
** ***R*_work_/*R*_free_	0.1933/0.2278
** **No. atoms	
** **Protein	1,330
** **Ligand/ion	5
** **Water	89
** ***B*-factors	
** **Protein	38.80
** **Ligand/ion	92.70
** **Water	39.40
** **R.m.s. deviations	
** **Bond lengths (Å)	0.003
** **Bond angles (°)	0.66
** **Ramachandran favoured (%)	98.7
** **Ramachandran outliers (%)	0.0
** **MolProbity Clashscore	0.36

R.m.s, root-mean square.

^*^Values in parentheses are for highest resolution shell.
