# Supplementary material for: Involvement of a eukaryotic-like ubiquitin-related modifier in the proteasome pathway of the archaeon Sulfolobus acidocaldarius
Source: Nat Commun. 2015 Sep 8;6:8163. doi: 10.1038/ncomms9163 (PMC4569737; doi:10.1038/ncomms9163)
Supplement: Supplementary Information — Supplementary Figures 1-9, Supplementary Tables 1-6, Supplementary Note, Supplementary Method and Supplementary References [file ncomms9163-s1.pdf]

Supplementary Materials

Supplementary Note

Supplementary Note:

Genomic context analysis of crenarchaeal *Urm1* homologues

In bacterial and archaeal organisms, genomic context analyses can often predict functional associations between the proteins encoded by the neighbouring genes (*1, 2*). The *S. acidocaldarius Urm1* (*Saci0669*) genomic locus was suggestive of an association between the modifier and several components of the proteasomal protein degradation pathway. Three genes, which encode a catalytic β–subunit of the 20S proteasome (*Saci0662*), the PAN proteasome regulatory ATPase (*Saci0656*), and a proteasome assembly chaperone (PAC2; *Saci0658*), respectively, were located within 11 kb of the *Urm1* gene (Supplementary Fig. 3A). This arrangement was conserved in a number of other *Sulfolobaceae* species. In addition, the β–subunit of the thermosome, an archaeal chaperonin, was detected at this genomic locus in all *Sulfolobaceae* species (Supplementary Fig. 3A), as was reported previously (*3*). This gene is also likely to be associated with proteasome pathways, as it has been shown that chaperonins play an important role in protein triage, channelling misfolded proteins to the proteasome for degradation (*4*). Finally, we observed that a gene encoding the fructose 1,6-bisphosphatase (FBP) glucose metabolism enzyme is harboured within the *Urm1* operon in the *Sulfolobales* family (Supplementary Fig. 3A). This arrangement is perhaps suggestive that gluconeogenesis is regulated by ubiquitin-like modification, and subsequent proteasomal degradation of this enzyme, as has been reported in yeast (*5*). *Urm1* genomic loci were also examined in more distantly related crenarchaeal species, where it was discovered that the *Urm1* gene was associated with either the thermosome β–subunit, or at least one of the 20S proteasome β–subunit, *PAC2* or *PAN* genes (Supplementary Fig. 3B to 3F). Furthermore, the *FBP* gene was also observed at the *Ignisphaera aggregans Urm1* locus (Supplementary Fig. 3B).

The genomic context analysis was also suggestive of an association between Urm1 and the archaeal ribosome. As reported previously (*3*), the *Urm1* operon also encodes the S17 subunit of the 30S ribosome in all of the crenarchaeal examples examined here (Supplementary Fig. 3A to 3F). In addition, the *Sulfolobaceae* family members, and the *I. aggregans* and *Fervidicoccus fontis* genomic environments contained a gene encoding an ATPase RNAaseL inhibitor (RLI); these proteins have previously been shown to play essential roles in ribosome biogenesis (*6*). Additionally, in *A. pernix*, *A. saccharovorans* and *C. lagunensis* the *Urm1* ORF was fused to a purine phospho-ribosyltransferase, which likely functions in nucleotide salvage pathways, and may play a role in ribosomal RNA turnover.

We also observed a linkage between Urm1 and tRNA modification in the genomic context analysis, consistent with the previously reported involvement of the *H. volcanii* SAMP2 in tRNA thiolation (*7*). A PHYRE2 search identified the *Igag0114* gene (Supplementary Table 5; below), located immediately next to the *Urm1* homologue in *I. aggregans*, as CGI121, a member of the KEOPS (kinase, putative endopeptidase and other proteins of small size) complex. Similarly, PHYRE2 searches were suggestive that the genes immediately upstream of *Urm1* in *S. solfataricus* and *S.acidocaldarius* (*Sso0284* and *Saci0668*, respectively) are CGI121 homologues (Supplementary Fig. 3A and B, and Supplementary Table 5). Interestingly, SAMP/CGI-121 fusions have been reported previously in the *Thaumarchaeal* kingdom (*3*), but CGI121 homologues were thought to be absent in the *Sulfolobales* (*3*). Both eukaryotic and archaeal KEOPS complexes have been shown to play key roles in tRNA modification (*8, 9*). We also noted the presence of a tRNA ribose methyltransferase at the *C. lagunensis Urm1* locus (*Calag1284*) (Supplementary Fig. 3F), providing further potential evidence of the role of urmylation in tRNA modification in the crenarchaea.

Finally, we noted a HerA/FtsK/VirB4/TrwB superfamily ATPase (*10, 11*) homologue close to the *Urm1* gene in *I. aggregans*, in addition to the *Sulfolobaceae* family homologue previously identified (*3*) (Supplementay Fig. 3A and B), implicating the urmylation pathway in DNA transport or cell division. Taken together, the genomic context analysis is therefore suggestive of a range of biological functions for the Urm1/SAMP pathway in archaeal cells. While these observations open several routes for further experimental investigation, we decided to pursue the possible roles of urmylation in the archaeal proteasomal degradation pathway in this study.

Supplementary Figures

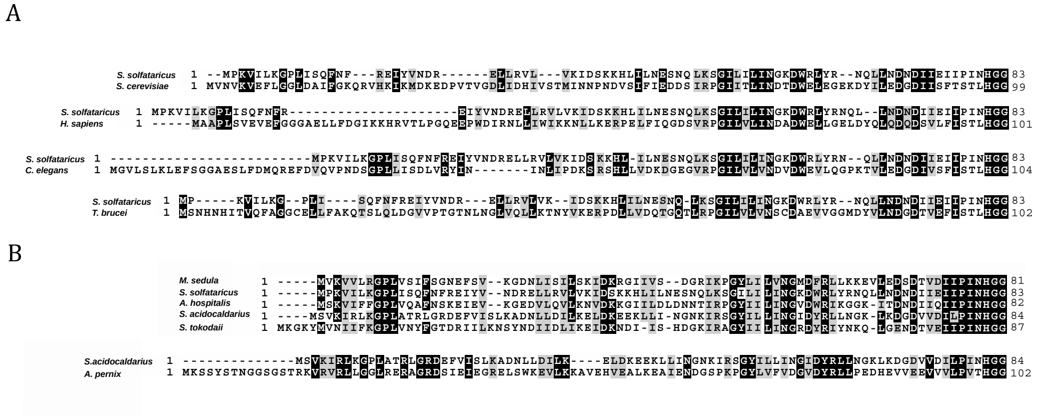

Supplementary Figure 1. Amino acid sequence conservation between archaeal and eukaryotic Urm1 proteins. (A) Sequence alignment of the *Sulfolobus solfataricus* Urm1 with eukaryotic homologues from budding yeast (*S. cerevisiae*), human (*H. sapiens*), worm (*C. elegans*) and trypanosome (*T. brucei*). (B) Sequence alignment of crenarchaeal Urm1 proteins from *Sulfolobus solfataricus*, *S. acidocaldarius*, *S. tokodaii*, *Metallosphaera sedula*, *Acidianus hospitalis* and *Aeropyrum pernix*. Identical and conserved residues are highlighted in black and grey, respectively.

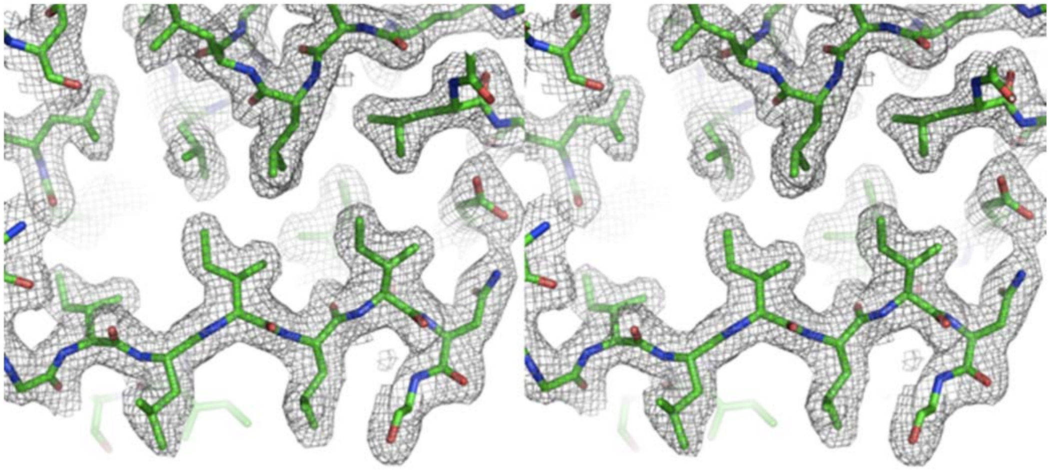

Supplementary Figure 2. Stereo image of a portion of the electron density map of the *S. solfataricus* Urm1 crystal structure. Secondary structure elements beta-3 and alpha-2 from chain A are shown [Type of map: 2Fo-Fc, Contour level: 1 sigma] (also see Figure 1A).

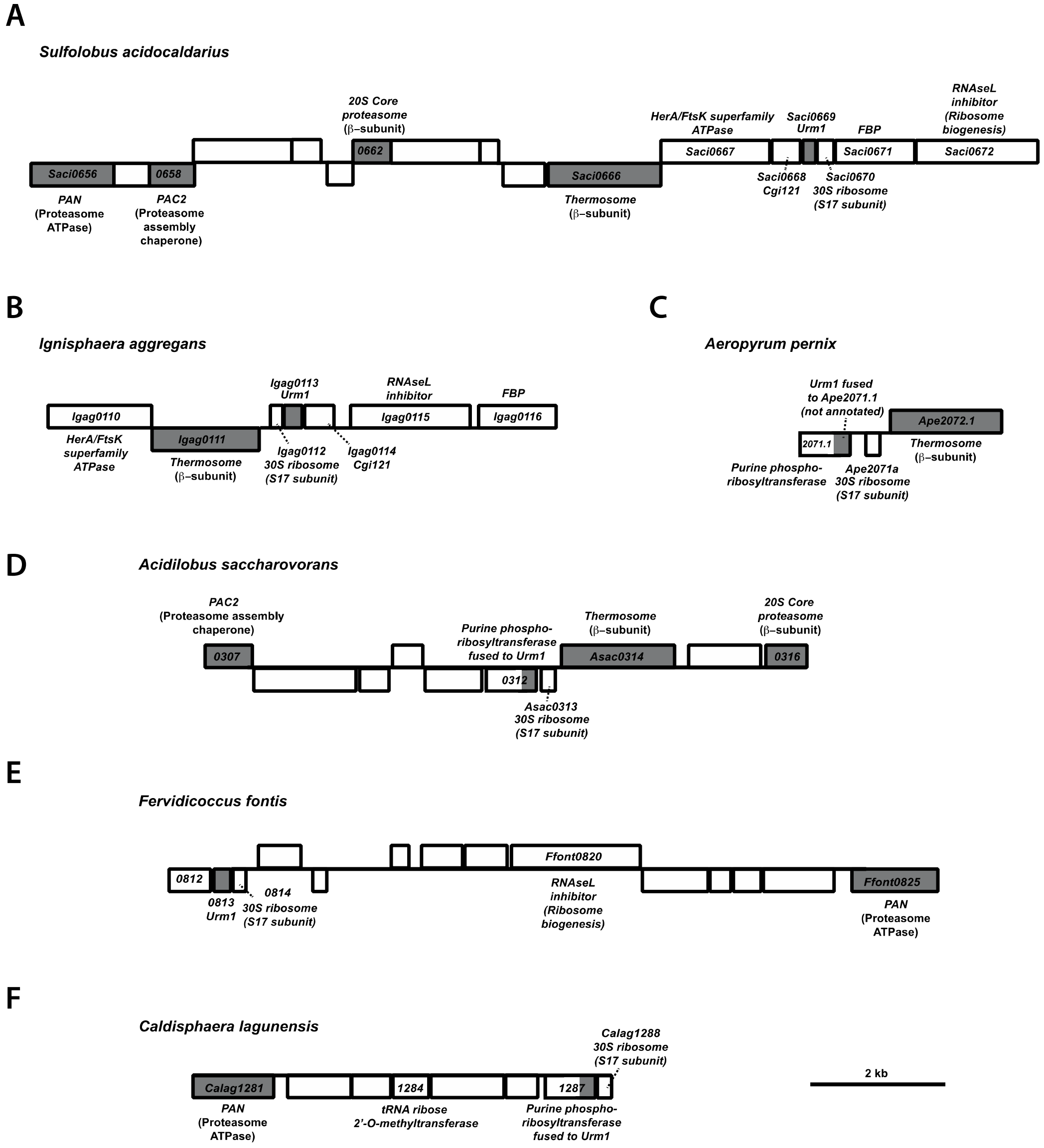

Supplementary Figure 3. Genomic context analyses of *Urm1* loci in crenarchaeal species are suggestive of an involvement of Urm1 modification in proteasomal pathways. (A) The *S. acidocaldarius Urm1* genomic locus encompasses the *Saci0656, Saci0658, Saci0662* and *Saci0666* genes (shaded grey) that are likely involved in proteasomal processing. (B) to (F) *Urm1* genomic loci in the crenarchaeal species *Ignisphaera aggregans*, *Aeropyrum pernix*, *Acidilobus saccharovorans*, *Fervidicoccus fontis* and *Caldisphaera lagunensis*, respectively. Details of the *Urm1* linkages to ribosome maturation and tRNA modification pathways are provided in the Supplementary Note (above). In all panels *Urm1* and the genes presumably involved in proteasomal pathways are shaded grey. ORFs positioned above the midline are transcribed left to right, and those below the line transcribed right to left. A putative homologue of CGI121; a member of the archaeal KEOPS complex (kinase, putative endopeptidase and other proteins of small size) is located in the Urm1/SAMP genomic locus in the crenarchaeal species *Ignisphaera aggregans* (Panels A and B; also see Supplementary Table 2C).

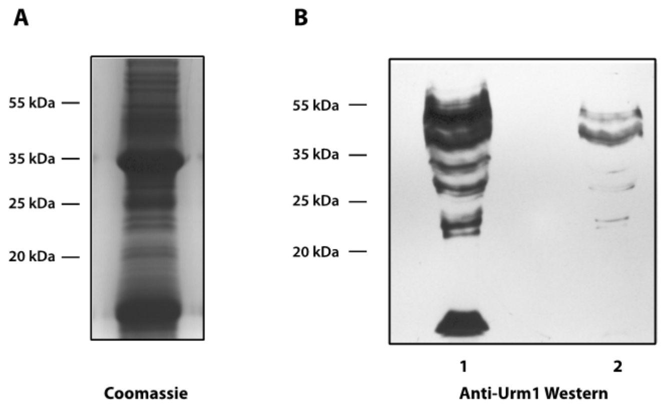

Supplementary Figure 4. SDS-PAGE polyacrylamide gels of the *in vitro* and *in vivo* generated Urm1 conjugates, subsequently analysed by GeLC tandem mass spectrometry. (A) *In vitro* urmylation of *S. acidocaldarius* cell free extract using N-terminally His-tagged Urm1 (band at approximately 10 kDa) and untagged ELSA/Uba4/ThiF E1-like enzyme (band at approximately 35 kDa), pulled own on Ni-NTA agarose, and run on a 15% SDS-PAGE gel. The reaction conditions, and Urm1-conjugate purification procedures, are described below in the Supplementary Methods. (B) *In vivo* overexpression of the N-terminally His-tagged Urm1 in the *S. acidocaldarius* strain MW001, run on a 15% SDS-PAGE gel. Western blot probed with anti-Urm1 antibody. Lane 1: Urm1 overexpression; lane 2: empty expression vector control. The transformation, overexpression and Urm1-conjugate purification procedures are described below in the Supplementary Methods.

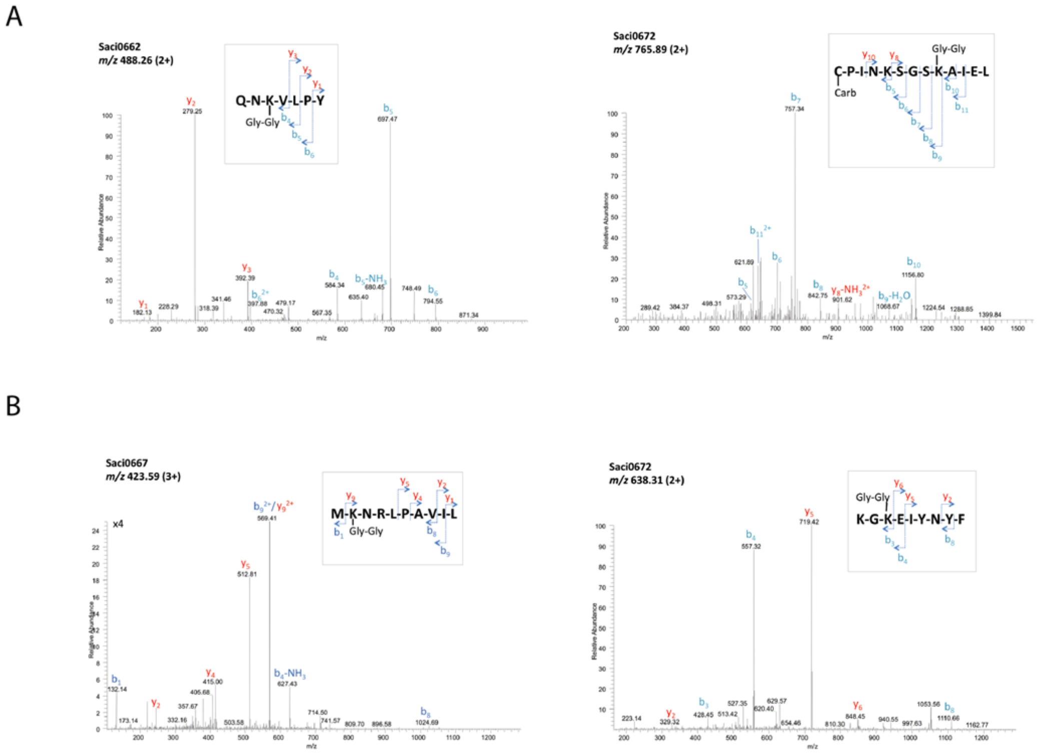

Supplementary Figure 5. Additional examples of MS/MS spectra of the diglycine modified peptides from the *in vitro* urmylation assay and *in vivo* Urm1 overexpression. (A) Saci0662 [Proteasome subunit β] and Saci0672 [RIL ATPase]) from the *in vitro* urmylation assay. (B) Saci0667 [HerA/FtsK/TrwB ATPase] and Saci0672 [RIL ATPase] from the *in vivo* Urm1 overexpression. *m/z* values of the precursor ions are shown above the top left of each spectrum. (2+) or (3+) indicates doubly or triply charged precursor ions, respectively. Spectra show the annotated peaks that are due to C-terminal y (coloured red) and N-terminal b (coloured blue) ions. In each case, the *m/z* values of the precursor ions and the *m/z* values of the fragment ions are consistent with diglycine-modified lysine residues. The amino-acid sequence of the chymotrypsin generated peptide, including the di-glycine modified lysine (denoted by 'Gly-Gly'), is shown within the grey box in each example. 'Carb' denotes a carbamidomethyl modification on a cysteine on the Saci0672 peptide shown in (A).

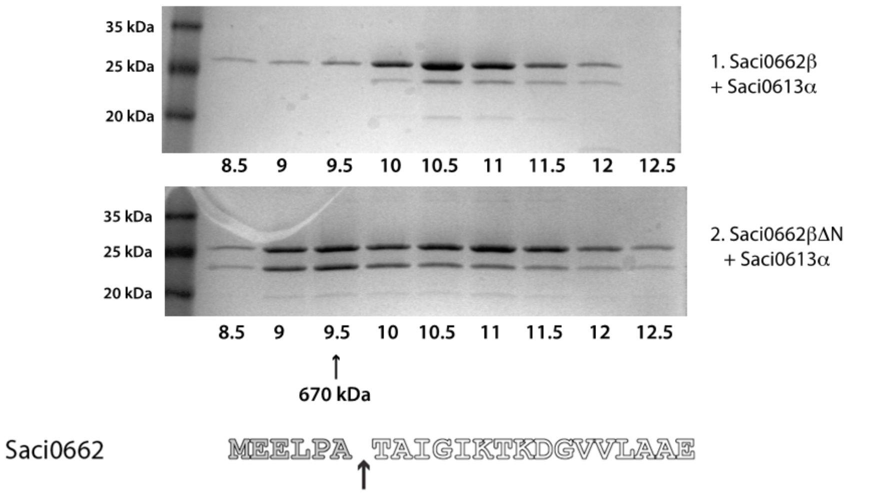

Supplementary Figure 6. Size exclusion chromatography analysis of the Saci0613/Saci0662 and Saci0613/Saci0662ΔN (deletion of N-terminal pro-peptide [MEELPA]) proteasomal complexes. Fractions eluted from the S200 HR 10/300 gel filtration column were resolved by SDS-PAGE. The 20S proteasome complex (≈ 660 kDa at a peak of 9.5 mls) was only stable when Saci0613 α-subunit was coexpressed with the N-terminally truncated Saci0662 β-subunit (Saci0662ΔN).

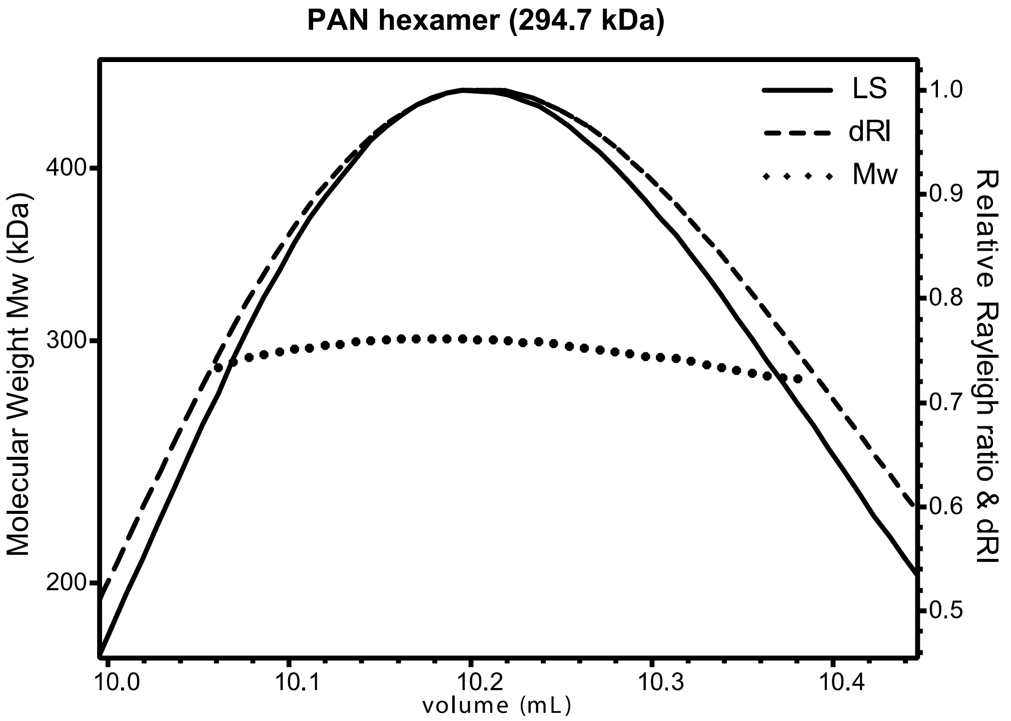

Supplementary Figure 7. SEC-MALS analysis of the PAN (proteasome activating nucleotidase) ATPase. The complex has a fitted molecular weight of 294.7 kDa (±0.069%), consistent with the predicted hexameric ring-shaped assembly, with a polydispersity of 1.000 (± 0.098%). Differential refractive index (dRI) and light scattering (LS) are plotted in conjunction with molecular weight (M_w).

A

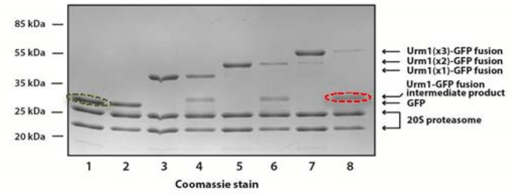

B

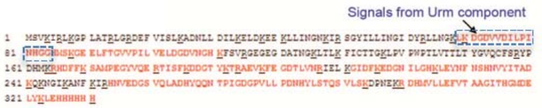

The matched peptides cover **57%** (189/331AA's) of the protein.

m/z Submitted	MH⁺ Matched	Intensity	Delta ppm	Modifications	Start	End	Missed Cleavages	Sequence
849.4540	849.4366	7504.0	20.5		165	170	1	(K)RHDFFK(S)
955.4500	955.4480	1022.0	2.15		187	194	1	(K)DDGTYKTR(A)
1050.5330	1050.5214	44630.0	11.0		199	207	0	(K)FEGDTLVNR(I)
1083.4960	1083.4980	4216.0	-1.82		324	331	0	(K)LEHHHHHH(-)
1266.5770	1266.5783	20160.0	-1.04		171	181	0	(K)SAMPEGYVQER(T)
1274.6370	1274.6263	2694.0	8.39		182	192	1	(R)TISFKDDGTYK(T)
1282.5610	1282.5732	31320.0	-9.53	1Oxidation	171	181	0	(K)SAMPEGYVQER(T)
1477.7620	1477.7645	13670.0	-1.71		195	207	1	(R)AEVKFEGDTLVNR(I)
1542.7760	1542.7911	11500.0	-9.78		212	225	1	(K)GIDFKEDGNILGHK(L)
1919.9170	1919.9280	3587.0	-5.72	1Oxidation	71	88	0	(K)DGDVVDILPINHGGHMSK(G)
1927.8920	1927.9185	43090.0	-13.7		226	241	0	(K)LEYNFNSHNVYITADK(Q)
2145.1180	2145.1121	3744.0	2.75		69	88	1	(K)LKDGDVVDILPINHGGHMSK(G)
2161.0940	2161.1070	10270.0	-6.02	1Oxidation	69	88	1	(K)LKDGDVVDILPINHGGHMSK(G)
2437.2610	2437.2609	18560.0	0.0281		89	111	0	(K)GEELFTGVVPILVELDGDVNGHK(F)
2590.3000	2590.2680	2562.0	12.4		301	323	0	(R)DHMVLLEFVTAAGITHGMDELYK(L)
2606.2940	2606.2629	4507.0	11.9	1Oxidation	301	323	0	(R)DHMVLLEFVTAAGITHGMDELYK(L)
2622.2800	2622.2578	4577.0	8.45	2Oxidation	301	323	0	(R)DHMVLLEFVTAAGITHGMDELYK(L)
2746.4160	2746.3691	588.0	17.1		300	323	1	(K)RDHMVLLEFVTAAGITHGMDELYK(L)
2762.4040	2762.3640	531.2	14.5	1Oxidation	300	323	1	(K)RDHMVLLEFVTAAGITHGMDELYK(L)
2778.3900	2778.3589	720.1	11.2	2Oxidation	300	323	1	(K)RDHMVLLEFVTAAGITHGMDELYK(L)
4486.1400	4486.1910	7042.0	-11.4		254	294	0	(R)HNVEDGSVQLADHYQQNTPIGDGPVLLPDNHYLSTQSVLSK(D)

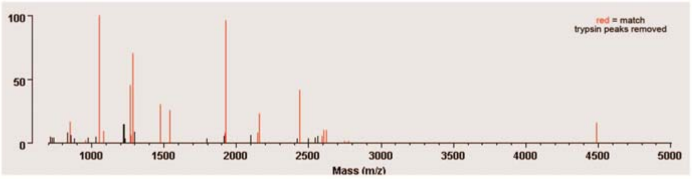

C

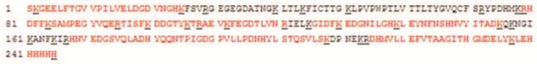

The matched peptides cover **69%** (170/245AA's) of the protein.

m/z Submitted	MH⁺ Matched	Intensity	Delta ppm	Modifications	Start	End	Missed Cleavages	Sequence
849.4410	849.4366	4110.0	5.18		79	84	1	(K)RHDFFK(S)
955.4330	955.4480	1809.0	-15.6		101	108	1	(K)DDGTYKTR(A)
1050.5290	1050.5214	36170.0	7.19		113	121	0	(K)FEGDTLVNR(I)
1083.4930	1083.4980	1932.0	-4.59		238	245	0	(K)LEHHHHHH(-)
1266.5790	1266.5783	22860.0	0.543		85	95	0	(K)SAMPEGYVQER(T)
1274.6370	1274.6263	1745.0	8.39		96	106	1	(R)TISFKDDGTYK(T)
1282.5670	1282.5732	22940.0	-4.86	1Oxidation	85	95	0	(K)SAMPEGYVQER(T)
1477.7700	1477.7645	11320.0	3.70		109	121	1	(R)AEVKFEGDTLVNR(I)
1542.7730	1542.7911	6383.0	-11.7		126	139	1	(K)GIDFKEDGNILGHK(L)
1927.9060	1927.9185	39970.0	-6.47		140	155	0	(K)LEYNFNSHNVYITADK(Q)
2437.2420	2437.2609	2268.0	-7.77		3	25	0	(K)GEELFTGVVPILVELDGDVNGHK(F)
2590.2470	2590.2680	5732.0	-8.11		215	237	0	(R)DHMVLLEFVTAAGITHGMDELYK(L)
2606.2500	2606.2629	7335.0	-4.96	1Oxidation	215	237	0	(R)DHMVLLEFVTAAGITHGMDELYK(L)
2622.2440	2622.2578	5517.0	-5.28	2Oxidation	215	237	0	(R)DHMVLLEFVTAAGITHGMDELYK(L)
2652.3510	2652.3879	14900.0	-13.9		1	25	1	(-)SKGEELFTGVVPILVELDGDVNGHK(F)
4486.0040	4486.1910	7366.0	-41.7		168	208	0	(R)HNVEDGSVQLADHYQQNTPIGDGPVLLPDNHYLSTQSVLSK(D)

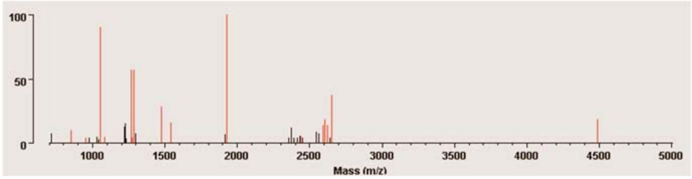

Supplementary Figure 8. Mass spectrometry analysis of the intermediate product generated during processing of an Urm1:GFP fusion protein. (A) Coomassie gel of the GFP and Urm1:GFP fusion protein processing assay described in Figure 5A (main text). The intermediate product (red circle) and a GFP only control (green circle) were excised from the gel, trypsin digested, and analysed by MALDI fingerprinting spectrometry. (B) MALDI fingerprinting analysis of the Urm1:GFP fusion intermediate. Amino acid sequences identified by the fingerprinting are coloured red. The blue arrow denotes a peptide representative of the C-terminus of Urm1. Peptide coverage is also observed for majority of the GFP amino-acid sequence, including peptides from both the N- and C-termini of GFP. The intermediate product is therefore representative of a short Urm1 C-terminal peptide (highlighted by the blue dotted box) fused to full length GFP, following almost complete degradation of the Urm1 tag. (C) Analysis of the GFP only control band.

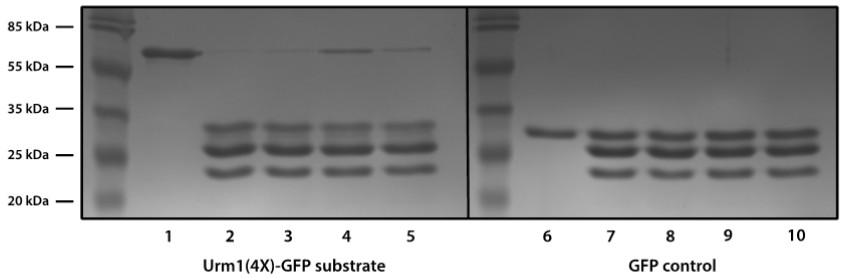

Supplementary Figure 9. AMP-PNP control assay confirming that the Urm1-GFP substrate processing by the 20S core proteasome is not mediated by a contaminating ATPase. Lane 1: Urm1(4X)-GFP substrate control without the 20S proteasome; Lane 2: Urm1(4X)-GFP plus the active 20S proteasome. Lanes 3, 4 and 5: as in lane 2, but with the addition of 3 µl 100 mM Tris (control), 3 µl 100 mM ATP (in 100 mM Tris), or 3 µl 100 mM AMP-PNP (in 100 mM Tris), respectively. The addition of non-hydrolysable AMP-PNP does not impair the processing reaction. Lanes 6-10; as in lanes 1-5, but using untagged GFP as a control. In all cases 10 µg of substrate was incubated with 20 µg of the proteasome complex for 1 hour at 70°C.

Supplementary Tables

A

Saci0669 Urm1	PDB	Confidence	% Identity	Organism	Description	Fold
1	1xo3	99.3	33	*Mus musculus*	Urm1	β-Grasp (ubiquitin-like)
2	1wgk	99.3	32	*Mus musculus*	Urm1	β-Grasp (ubiquitin-like)
3	2gjl	99.2	30	*Saccharomyces cerevisiae*	Urm1	β-Grasp (ubiquitin-like)
4	2k9x	98.8	26	*Trypanosoma brucei*	Urm1	β-Grasp (ubiquitin-like)

B

Sso5559 Urm1	PDB	Confidence	% Identity	Organism	Description	Fold
1	1wgk	98.8	32	*Mus musculus*	Urm1	β-Grasp (ubiquitin-like)
2	1xo3	98.8	32	*Mus musculus*	Urm1	β-Grasp (ubiquitin-like)
3	2gjl	98.6	34	*Saccharomyces cerevisiae*	Urm1	β-Grasp (ubiquitin-like)
4	2k9x	98.4	30	*Trypanosoma brucei*	Urm1	β-Grasp (ubiquitin-like)

Supplementary Table 1. PHYRE2 (Protein Homology/analogY Recognition Engine V 2.0) searches predict eukaryotic Urm1 proteins as the closest structural homologues of the *S. acidocaldarius* (A) and *S. solfataricus* (B) Urm1/SAMP proteins. Searches were performed on the PHYRE2 protein fold recognition server (http://www.sbg.bio.ic.ac.uk/phyre2/html/page.cgi?id=index).

A

No:	PDB	Alignment Length	P-value	% Identity	Organism	Description
1	1v8c	64	0.0002	18.8	*Thermus thermophilus*	MoaD-related protein
2	2qjl	63	10e-4.7	31.7	*Saccharomyces cerevisiae*	Ubiquitin-related modifier (Urm1)
3	4n6e	62	10e-5.4	19.4	*Amycolatopsis orientalis*	CysO/BexX homologue
4	3dwg	61	10e-5.0	21.3	*Mycobacterium tuberculosis*	CysO homologue
5	2ax5	61	0.001	32.8	*Saccharomyces cerevisiae*	Ubiquitin-related modifier (Urm1)
6	1xo3	60	10e-5.7	36.7	*Mus musculus*	Ubiquitin-related modifier (Urm1)
7	3po0	58	10e-4.9	20.7	*Haloferax volcanii*	SAMP1 homologue
8	4hro	58	0.0003	20.7	*Haloferax volcanii*	SAMP1 homologue

B

No:	PDB	Z-score	% Identity	Organism	Description
1	1v8c	11.9	14	*Thermus thermophilus*	MoaD-related protein
2	2l52	10.8	31	*Methanosarcina acetivorans*	SAMP1 homologue
3	2qjl	10.7	28	*Saccharomyces cerevisiae*	Ubiquitin-related modifier (Urm1)
4	3po0	10.6	16	*Haloferax volcanii*	SAMP1 homologue
5	2pko	10.6	28	*Saccharomyces cerevisiae*	Ubiquitin-related modifier (Urm1)
6	4n6e	10	17	*Amycolatopsis orientalis*	CysO/BexX homologue
7	3dwg	9.7	17	*Mycobacterium tuberculosis*	CysO homologue
8	2ax5	9.2	27	*Saccharomyces cerevisiae*	Ubiquitin-related modifier (Urm1)

Supplementary Table 2. Structural homologues of the *S. solfataricus* Urm1 protein, and genomic linkage between the *Urm1* and *CGI121* genes. (A) Urm1 homologues identified by VAST (Vector Alignment Search Tool) search (*12*). (B) Urm1 homologues identified by Dali search (*13, 14*).

A

ORF	Protein Description	Expect Value	Peptides
Saci2271	Carbon-monoxide dehydrogenase	0.00097	L.GIKGKLL.L
Saci0179	E1-like enzyme	0.00027	F.LLNDVAVKL.R
		0.014	L.GVQEKISQL.K
		0.00074	L.KIAIVGCGAL.G
		4.00E-06	L.VLGLGVQEKISQL.K
Saci1355	Phosphoglycerate kinase	0.031	L.KIIENL.I
Saci0839	Reverse gyrase	0.063	L.ITKDQEVIY.I
Saci1875	CRISPR-associated protein CscA	0.0066	Y.GSGSEDNLNNVKENL.N
Saci1020	Aldehyde oxidase	0.021	F.IALEKLL.K
Saci0662	20S core proteasome (β-subunit)	0.039	Y.QNKVLPY.I
Saci1417	Phosphoenolpyruvate synthase	0.073	F.LNVSKNDLF.S
*Saci1149**	*Acetyl-CoA synthetase**	0.048	L.GGNSLKPLMY.L
		0.0073	Y.QDLLIRANKL.I
		0.019	W.ICGSLNSISKGML.F
Saci0669	Urm1/SAMP	0.015	L.KADNLLDIL.K
		0.0042	L.NGKLKDGDVVDILPINHGG
Saci0862	tRNA synthetase	0.013	F.IKDNSDF.V
Saci0671	Fructose 1-6-bisphosphatase	0.051	L.AEAKSQSLL.I
		0.032	F.KEAAKVAKEL.G
*Saci0672**	*RNAaseL inhibitor (RLI) ATPase**	0.042	F.CPINKSGSKAIEL.S
Saci2229	Thiamine pyrophosphate	0.0074	Y.NKTPLVL.L
Saci1255	Predicted flavoprotein (COG0431)	0.016	L.KNAIDW.A
Saci0051	Rad50 DNA DSB-repair ATPase	0.0095	L.KSLVSL.E
Saci0046	BPS2 SMC-family ATPase	0.0039	L.KGKANIEL.K
		0.024	L.STSEKTTLALIL.I
Saci1109	3-hydroxybutyryl-CoA dehydrogenase	0.024	L.GVKSGKGFY.T
*Saci1690**	*Alcohol-dehydrogenase**	0.017	L.KDFGADL.V
Saci0422	NTP-transferase	0.044	Y.KEAVSLF.D
Saci0080	Ribosomal protein S13 (COG0099)	0.0055	L.NNKIIPESPNW.I
Saci1838	Carbon monoxide dehydrogenase	0.00011	Y.KAEDVAEEVSIDY.E
Saci0659	Queuine tRNA-ribosyltransferase	0.03	Y.IGKEEDL.H
Saci2176	Unknown function	0.012	W.IVGRNLNKVL.E
Saci0581	Ribosomal protein L6P/L9E (COG0097)	0.0038	Y.KKEVIGL.E
Saci1611	Glutamine amidotransferase	0.014	L.VVLTNKGEL.I
Saci0576	Ribosomal protein L30/L7E (COG1841)	0.037	F.VESIKDGKL.R
Saci0797	Unknown function	0.03	Y.ELKLEIQNMQKVIENL.N
Saci0658	Proteasome Chaperone (PAC2)	0.0035	L.MEKQLL.M

B

ORF	Protein Description	Expect Value	Peptides
Saci2271	Carbon monoxide dehydrogenase	0.012	L.GIKGKLLL.D
		0.0011	L.KVNGIVAVF.S
Saci0685	Translation Elongation factor Tu	0.045	L.KVGDKIVF.M
		0.00076	L.VVSAKKGEY.E
Saci1455	Alanyl-tRNA synthetase	0.0084	Y.ARQQDEKL.N
Saci1767	Fumarylacetoacetate (FAA) hydrolase	0.012	W.IVTKDEIDNINNL.R
		0.0001	L.SNGITLKPGDVISTGTPSGVAL.A
Saci0051	Rad50	0.0041	L.KSLVSL.E
		3.5e-06	Y.LKNSDILEEDIQEQEQRL.N
*Saci1690**	*Alcohol-dehydrogenase**	0.036	L.AFDKNGL.N
Saci1572	Leucyl-tRNA synthetase	0.0047	Y.VEELKPL.V
*Saci1149**	*Acetyl-CoA synthetase**	0.069	L.DKLNGVLF.H
Saci1020	Aldehyde oxidase	0.022	F.IALEKLL.K
Saci1849	Beta-galactosidase	0.044	F.HDEAEKIGL.N
Saci0386	Unknown function	0.017	Y.ENAGMGSKEATMDIIL.N
		4.6e-07	Y.NLKPLDEAEIIGIIDEEKKEVEQY.E
Saci1417	Phosphoenolpyruvate synthase	0.032	F.LNVSKNDLF.S
Saci2218	Conserved 3-hydroxybutyryl-CoA	0.002	L.DQFTKPDAIL.A
Saci1282	Phosphoribosylpyrophosphate synthetase	0.011	F.VLAPDKGAL.N
Saci2017	Phosphohistidine phosphatase/mutase	0.00029	Y.QSAEVILDELGEDQGKIETL.N
Saci0658	Proteasome Chaperone (PAC2)	0.018	L.MEKQL.L
Saci1024	Serine/trypsin protease	0.0085	L.TKQITL.D
Saci0083	DNA-directed RNA polymerase (α subunit)	0.045	L.SELKSKL.E
Saci0612	Shwachman-Bodian-Diamond syndrome	9.80E-05	L.EIPAGAQQDVIDKL.N
	protein (exosome-related)	4.10E-06	L.IAELEIPAGAQQDVIDKL.N
Saci0690	50S ribosomal protein L30e	0.00077	F.EGELKTLL.R
*Saci0672**	*RNAaseL inhibitor (RLI) ATPase**	0.031	F.KGKEIYNYF.Q
Saci0576	50S ribosomal protein L30P	0.0047	F.VESIKDGKL.R
Saci0667	HerA/FTSK/TrwB ATPase	0.0018	MKNRLPAVIL.L
Saci0669	Urm1/SAMP	0.0095	L.KADNLL.D
		0.016	L.KELDKEEKL.L
Saci0656	Proteasome Activating Nucleotidase (PAN)	0.0066	Y.DETLVKLL.E

Supplementary Table 3. Urm1 modified substrates identified by mass spectrometry. (A) Glycine-glycine modified peptides, following *in vitro* urmylation of *S. acidocaldarius* cell-free extract (using recombinant Urm1 and ELSA/Uba4p/ThiF proteins), identified by GelC tandem mass spectrometry. (B) Glycine-glycine modified peptides generated *in vivo* following Urm1 overexpression in *S. acidocaldarius* cells, identified by GelC tandem mass spectrometry. Protein identities were determined by searching against the MUTAGEN *Sulfolobus* database (2013) http://www.sulfolobus.org/cbin/mutagen.pl?page=main. Full experimental details are described in the Methods. Modified lysines are underlined. Peptides that are modified on identical lysine residues in both the *in vitro* and *in vivo* approaches are highlighted in white lettering on a black background, while proteins that are modified in both the approaches, but on differing peptides, are marked with an asterisk. The expect value, or E-value, is the number of matches with equal or better scores that are expected to occur by chance alone. It is directly equivalent to the E-value in a Blast search result (http://www.ncbi.nlm.nih.gov/BLAST/tutorial/Altschul-1.html#head2). For a score that is exactly on the default significance threshold, ($p<0.05$), the expectation value is also 0.05.

S. acidocaldarius subunit	*S. cerevisiae* homologue	Eukaryotic proteasome subunit homologue	Alignment region (*S. acidocaldarius)*	Alignment region (*S. cerevisae)*	% identity	Positives	E Value
Saci0909 (catalytic)	YPR103W	β-type 5 (catalytic)	6 to 192	71 to 259	31	103/189	1.00E-19
Saci0662 (structural)	YOR157C	β-type 7 (structural)	7 to 183	31 to 207	31	100/180	3.00E-21

Supplementary Table 4. PSI-BLAST searches reveal the closest *S . cerevisiae* homologues of the Saci0909 (catalytic) and Saci0662 (structural) *S.acidocaldarius* proteasome β–subunits, respectively. PSI-BLAST searches were performed at http://www.ncbi.nlm.nih.gov/blast.

Igag0114	PDB	Confidence	% Identity	Organism	Description	Fold
1	1dz0	95.2	26	*Pyrococcus furiosus*	CGI121	PF0523-like

Sso0284	PDB	Confidence	% Identity	Organism	Description	Fold
1	1dz0	34.5	16	*Pyrococcus furiosus*	CGI121	PF0523-like

Sso0668	PDB	Confidence	% Identity	Organism	Description	Fold
9	3enh	15.8	21	*Methanocaldococcus jannaschii*	CGI121	CGI121-like

Supplementary Table 5. PHYRE2 searches predict CGI121 homologues (a member of the KEOPS complex) next to the *Urm1* gene in crenarchaeal genomes. Searches with *Ignisphaera aggregans* Igag0114 and *S. solfataricus* Sso0284 identify CGI121 homologues as the top hit. Supplementary Figure 3A and B (above) illustrates the genomic linkage between the *Urm1* and *CGI121* genes in both species, respectively. Searches were performed on the PHYRE2 protein fold recognition server (http://www.sbg.bio.ic.ac.uk/phyre2/html/page.cgi?id=index).

Primer	Sequence (5' - 3')
Sso5559Urm1for	GCGCATATGCCAAAAGTGATCTTGAAAGGACC
Sso5559Urm1rev	GCGCTCGAGACCACCGTGATTTATTGGTATTATTTC
Saci0669Urm1for	GCGCATATGAGTGTGAAGATAAGGCTAAAAGGTCCTCTGG
Saci0669Urm1XhoIrev	GCGCTCGAGTTAGCCACCATGATTTATTGGTAAAATATCTAC
Saci0669Urm1NdeIrev	GCGCATATGGCCACCATGATTTATTGGTAAAATATCTAC
Saci0179Uba4/ThiFfor	GCGCATATGGAAAGATATGCTAGACAATTACTTGTATTGG
Saci0179Uba4/ThiFrev	GCGCTCGAGTTATCTATTAGACTTTTTAAAGCATTTTCCACTGG
Saci0666ThermBfor	GCGCATATGTCAGCTACAGCTACAGTTGCTACAACACC
Saci0666ThermBrev	GCGCTCGAGGTCTTCTTCTTTACCTTTTTCAGACTC
Saci0656PANfor	GCGCATATGTTCGGGGATGTTGATAGTTATAAGAATCGTG
Saci0656PANrev	GCGCTCGAGTAAGTATTTCTCGGTTCTTGTATTAATCGCTTTCC
Saci0613ProtAfor	GCGCATATGGCTTTAGGTCCAGCTGCGATGGGATATG
Saci0613ProtArev	GCGCTCGAGTTATATCTTTTGCAAAAGATCTGACCTTTCTTC
Saci0662ProtBFLfor	GCGCATATGGAGGAATTACCCGCTACTGCTATAGGAATAAAG
Saci0662ProtBCLIPfor	GCGCATATGACTGCTATAGGAATAAAGACCAAGGATGGTGTGG
Saci0662ProtBrev	GCGCTCGAGAATTTTTATGAAATCTTCATAGATATTGTTTTTG
Saci0909ProtBFLfor	GCGCATATGAATCTTCAAAATAAAATTCTGAAGGGAACAAC
Saci0909ProtBCLIPfor	GCGCATATGACGACGACCGTAGGGATTAAGGTCAAGGATGGAG
Saci0909ProtBrev	GCGCTCGAGTCTCTTAGTGATATAATATTCTTTCTCTTCATG
Saci0671FBPfor	GCGCATATGATGAAAACAACAATTAGTGTAATAAAAGCAGATATAGG
Saci0671FBPrev	GCGCTCGAGATCTCCTGATGGTCCGCTTTTTTCTTTTGAATATATAC
Urm1FBPminusATGmut	GATATTTTACCAATAAATCATGGTGGCCATAAAACAACAATTAGTGTAATAAAAGCAG
sfGFPfor	GCGCATATGAGCAAAGGAGAAGAACTTTTCACTGGAGTTG
sfGFPrev	GCGCTCGAGTTTGTAGAGCTCATCCATGCCATGTGTAATCC

Supplementary Table 6. Oligonucleotides used in the generation of *E. coli* expression constructs used in this study (*Nde*I and *Xho*I restriction endonuclease sites used for cloning are underlined).

Supplementary Materials and Methods

Expression constructs

Sulfolobus solfataricus urm1 (Sso5559) and *S. acidocaldarius Uba4/ThiF (Saci0179), thermosome β–subunit (Saci0666), FBP (Saci0671), PAN (Saci0656),* genes were amplified by PCR from *S. solfataricus* P2 or *S. acidocaldarius* DSM639 genomic DNA, respectively, using the primers described in Supplementary Table 6 (above). PCR products were cloned individually into pET30a (Novagen) using *Nde*I and *Xho*I sites, positioning the ORFs in frame with a C-terminal hexa-histidine tag. In addition, the *S. acidocaldarius urm1 (Saci0669)* ORF was amplified, using the primers Saci0669Urm1for and Saci0669Urm1XhoIrev, and cloned into pET28a (Novagen) using *Nde*I and *Xho*I sites, positioning the ORF in frame with a N-terminal, thrombin cleavable, hexa-histidine tag. The Superfolder (sf) GFP gene was amplified by PCR from a commercial clone (Sandia Biotech [product no. 23004006]), using the primers indicated in Supplementary Table 6 (above), and cloned into pET30a using *Nde*I and *Xho*I sites, positioning the ORFs in frame with a C-terminal hexa-histidine tag. N-terminal Urm1 fusion proteins of either FBP or sfGFP, were generated by cloning *urm1* (amplified with the primers Saci0669Urm1for, and Saci0669Urm1NdeIrev) into the *Nde*I site of the FBP:pET30a and sfGFP:pET30 constructs. Site-directed mutagenesis (QuikChange, Stragene), using the primer Urm1FBPminusATG, was performed to remove the FBP start methionine from the resultant Urm1:FBP fusion protein. The proteasome-α *(Saci0613)* ORF was amplified using the primers indicated in Supplementary Table 6 (above), and cloned using *Nde*I and *Xho*I sites into pET-DUET (Novagen) to generate an untagged construct. Full-length, and N-terminally truncated proteasome-β1 *(Saci0662)* and β2 *(Saci0909)* ORFs were amplified using the primers indicated in Supplementary Table 6 (above), and cloned using *Nde*I and *Xho*I sites into pET30a and pCDF-DUET (Novagen), respectively, to generate C-terminally His-tagged or S-tagged constructs, respectively. In all cases, the clones were verified by DNA sequencing of the complete ORF.

Protein Expression and Purification

C-terminally His-tagged *S. solfataricus* Urm1, and *S. acidocaldarius* PAN, thermosome β–subunit, FBP, Urm1-FBP fusion, GFP and Urm1-GFP fusion proteins were expressed in Rosetta (DE3) pLysS *E. coli* cells (Novagen). Cultures were grown at 37 °C to an OD_{600nm} of 0.6, then the temperature was lowered to 20°C, and the cells induced overnight with 0.33 mM IPTG. Cells were harvested by centrifugation, resuspended in 20 mM Tris [pH 8.0], 300 mM NaCl, 5% glycerol, 1 mM DTT, plus 1X EDTA-free protease inhibitors (cOmplete cocktail; Roche), and lysed by sonication. Insoluble material was removed via centrifugation (17 000 x *g* for 10 mins). The soluble fraction was subsequently heat clarified at 70°C for 20 minutes and centrifuged (17 000 x *g* for 10 mins) again to remove insoluble material. The supernatants were then applied by gravity flow to a column containing a 2.5 ml bed volume of Ni-NTA agarose (Qiagen). The column was washed with 20 mM Tris [pH 8.0], 300 mM NaCl, 5% glycerol, 1 mM DTT, plus 15 mM imidazole, and the proteins eluted with the same buffer plus 500 mM imidazole. Fractions containing the purified proteins were pooled and concentrated before running a final size-exclusion purification step over a Superdex 200 16/600 column (GE Healthcare), or an S75 16/600 column (GE Healthcare) for the Urm1 protein, in 20 mM Tris [pH 8.0], 300

mM NaCl, 5% glycerol, 1 mM DTT. Fractions containing the purified proteins were pooled, concentrated, aliquoted and flash frozen in liquid N_2. Protein concentrations were quantified by UV spectrophotometry.

N-terminally His-tagged and untagged *S. acidocaldarius* Urm1 (Saci0669) proteins were expressed and purified as described above, but prior to the final size-exclusion chromatography step, half of the tagged protein was dialysed in thrombin cleavage buffer (20 mM Tris-HCl [pH 8.0], 150 mM NaCl, 2.5 mM $CaCl_2$, 5% glycerol) overnight at R/T. The N-terminal His-tag was then cleaved by the addition of 25 units of thrombin (Novagen) at R/T for 3 hours. The cleaved sample was then applied by gravity flow to a column containing a 2.5 ml bed volume of Ni-NTA agarose (Qiagen), and washed with 20 mM Tris [pH 8.0], 300 mM NaCl, 5% glycerol, 1 mM DTT, plus 15 mM imidazole. Finally, both the His-tagged and untagged Urm1 proteins were purified further by size-exclusion chromatography over a Superdex 75 16/600 column, as described above.

Untagged ELSA/Uba4p/ThiF (Saci0179) was expressed and purified as described above, but after the heat clarification stage, 500 units of Benzonase nuclease (Sigma) were added and the NaCl concentration was reduced to 50 mM before loading onto a HiTrap QFF column (GE Healthcare). The proteins were eluted from the QFF column with a gradient of 50-1000 mM NaCl, in 20 mM Tris pH 8.0, 5% glycerol, 1 mM DTT. Fractions containing the purified protein was pooled and precipitated with 1.15 M $(NH_4)_2SO_4$. Following centrifugation (17 000 x g for 10 mins) the $(NH_4)_2SO_4$ cut pellet was resuspended in 20 mM Tris [pH 8.0], 300 mM NaCl, 5 % glycerol, 1 mM DTT and purified further by size-exclusion chromatography over a Superdex 75 16/600 column. Fractions containing the purified proteins were pooled, concentrated, aliquoted, and flash frozen in liquid N_2. Protein concentrations were quantified by UV spectrophotometry.

The *S. acidocaldarius* 20S core proteasome was reconstituted by coexpressing the untagged Saci0613 α-subunit with the N-terminally truncated, C-terminally His-tagged Saci0662 β-subunit, either with or without coexpression of the N-terminally truncated Saci0909 catalytic β-subunit, in Rosetta (DE3) pLysS *E. coli* cells. Cells were grown at 37°C to an OD_{600nm} of 0.6, then the temperature was lowered to 20°C, and the cells induced overnight with 0.33 mM IPTG. Cells were harvested by centrifugation, resuspended in 20 mM Tris [pH 8.0], 300 mM NaCl, 5% glycerol, 1 mM DTT, without protease inhibition, and lysed by sonication. The 20S proteasome complexes were then purified by heat clarification, Ni-NTA agarose IMAC, and size-exclusion chromatography over a Superdex 200 16/600 column, as described above.

Crystallisation and X-ray structure determination

Crystals of *Sulfolobus solfataricus* Urm1 (*Sso* Urm1) were grown by hanging drop vapour diffusion, in which equal volumes of Urm1-His_6 protein (25 $mg.ml^{-1}$) and reservoir solution (30% PEG 4000, 0.2 M ammonium sulphate) were mixed and allowed to equilibrate against 70 µl reservoir solution. Crystals appeared within 3-4 days at 19°C in a Formulatrix Imager. Crystals were transferred to a drop consisting of crystallisation solution supplemented with 25% glycerol, and subsequently flash frozen in liquid nitrogen.

X-ray diffraction data were collected to 2.2 Å resolution on a Bruker X8 Proteum diffraction system, comprising a MICROSTAR microfocus rotating anode X-ray generator equipped with HELIOS multilayer X-ray optics and a PLATINUM135 CCD detector. *These experiments were performed in the Crystallographic X-ray facility at the Department of Biochemistry, University of Cambridge*. The data were integrated, scaled and merged using the PROTEUM2 data processing software (*15, 16*) and Xprep (*15, 17*). *Sso* Urm1 crystallised in the orthorhombic space group P 2_1 2_1 2_1, with unit cell dimensions a = 31.49 Å, b = 65.19 Å, c = 109.36 Å, and two molecules in the asymmetric unit.

The structure was determined using the BALBES automated molecular replacement pipeline (*18*). The crystallographic model was completed using alternating cycles of manual rebuilding in Coot (*19*) and refinement with Phenix Refine (*20*), yielding a Molprobity clashscore (*21*) of 0.36 and final R-work and R-free values of 0.1933 and 0.2278, respectively. 98.7% of residues fall within the favoured region of the Ramachandran plot, and there are no Ramachandran outliers.

The following amino acids were omitted from the final model due to weak or absent electron density: chain A: residues 1 and 81-83; chain B: residues 1 and 80-83. Details of data processing and crystallographic refinement can be found in Table 1 of the article.

In vitro **urmylation of** ***S. acidocaldarius*** **cell extract for mass-spectrometry (MS) analysis**

S. acidocaldarius cell-free extracts were prepared by harvesting 1 L of cells at OD_{600nm} at 0.6, washed in 1xTBS (10 mM Tris [pH 8.0], 150 mM NaCl), and resuspended in 5 ml 1xTBS, 0.1% Triton-X-100, 0.1% β-mercaptoethanol, and 1X EDTA-free protease inhibitors (Roche). Cells were disrupted by sonication and the lysate clarified by centrifugation. 250 µl of the cell-free extract was added to 500 µl urmylation reaction buffer, supplemented with 375 µg N-terminally His-tagged Urm1 protein, 375 µg Uba4p/ThiF enzyme and 10 mM ATP, and incubated for 1.5 hours at 70°C. Urmylated products were purified by Ni-NTA pulldown, washed in TBST (10 mM Tris [pH 8.0], 150 mM NaCl, 0.1% Tween20). The products were eluted by boiling in 2X laemmli protein loading buffer, separated by SDS-PAGE, and analysed by GeLC-MS/MS.

In vivo **overexpression of Urm1 for mass-spectrometry analysis**

N-terminally hexa-his tagged Saci0669 (Urm1) was overexpressed in *Sulfolobus acidocaldarius* strain MW001 using the vector pCMalLacS (*22*). The ORF encoding the N-terminally His-tagged *S. acidocaldarius* Urm1 (cloned into vector pET28a, as described above) was subsequently PCR amplified using the primers JB157_F (aaaaaccATGGGCAGCAGCCATCATCATC) and JB157_R (aaaaacggccgTTAGCCACCATGATTTATTGGTAAAATATC) and subcloned into pCMalLacS using *Nco*I and *Eag*I restriction sites. The resultant N-terminal hexa-his Urm1 overexpression vector was methylated, electroporated into competent MW001 *Sulfolobus acidocaldarius* cells, and plated onto solid media lacking uracil as described in (*23*). Single colonies were isolated, and cultures were

established and induced for Urm1 expression in Brock's media containing 0.4% maltose as described in (*23*), at an OD_{600} of 0.06. Cells were grown following induction at 75°C with shaking, to an OD_{600} of 0.7. Cells were harvested by centrifugation at 4000 *g*, resuspended in 10 ml 1X TBS (10 mM Tris-HCl [pH8], 150 mM NaCl), plus 1X EDTA-free protease inhibitors (Roche), and snap frozen in liquid nitrogen. The cells were then defrosted and disrupted by sonication, and the cell extract was then clarified by centrifugation (18000 *g* for 20 minutes at 4°C). The soluble lysate was subsequently loaded onto 2.5 ml bed volume Ni-NTA agarose prewashed in 1X TBS. The column was then washed with 1X TBS plus 15 mM imidazole and finally the His-tagged conjugates were eluted from the column with 1X TBS plus 500 mM imidazole. The elutant was then concentrated with ultrafiltration spin columns (3000 MWCO Vivaspin, Sartorius) down to a final volume of 100 µl, and added to an equal volume of 2X laemmli protein loading dye. Products were then resolved by SDS-PAGE on 15% polyacrylamide gels. Overexpression of Urm1 was confirmed by Western blot analysis of duplicated gels probed with an anti-Urm1 specific antibody (see Supplementary Figure 4).

Mass Spectrometry and MS data analysis

Sets of 12 gel slices per lane from 1D gels (15% PAGE gels) were excised and transferred into a 96-well PCR plate. The gel bands were cut into $1mm^2$ pieces, destained, reduced (DTT) and alkylated (iodoacetamide) and subjected to enzymatic digestion with chymotrypsin overnight at 30°C. After digestion, the supernatant was pipetted into a sample vial and loaded onto an autosampler for automated LC-MS/MS analysis.

All LC-MS/MS experiments were performed using a nanoAcquity UPLC (Waters Corp., Milford, MA) system and an LTQ Orbitrap Velos hybrid ion trap mass spectrometer (Thermo Scientific, Waltham, MA). Separation of peptides was performed by reverse-phase chromatography using a Waters reverse-phase nano column (BEH C18, 75 mm i.d. x 250 mm, 1.7 mm particle size) at flow rate of 300 nL/min. Peptides were initially loaded onto a pre-column (Waters UPLC Trap Symmetry C18, 180 mm i.d x 20mm, 5 mm particle size) from the nanoAcquity sample manager with 0.1% formic acid for 3 minutes at a flow rate of 5 mL/min. After this period, the column valve was switched to allow the elution of peptides from the pre-column onto the analytical column. Solvent A was water + 0.1% formic acid and solvent B was acetonitrile + 0.1% formic acid. The linear gradient employed was 5-40% B in 60 minutes.

The LC eluant was sprayed into the mass spectrometer by means of a New Objective nanospray source. All *m/z* values of eluting ions were measured in the Orbitrap Velos mass analyzer, set at a resolution of 30000. Data dependent scans (Top 20) were employed to automatically isolate and generate fragment ions by collision-induced dissociation in the linear ion trap, resulting in the generation of MS/MS spectra. Ions with charge states of 2+ and above were selected for fragmentation. Post-run, the data was processed using Protein Discoverer (version 1.3., ThermoFisher). Briefly, all MS/MS data were converted to mgf files and twelve files (representing the entire gel lane) were combined and submitted to the Mascot search algorithm (Matrix

Science, London UK) and searched against a *Sulfolobus acidocaldarius* database (2367 sequences; 652117 residues, http://www.sulfolobus.org) using a fixed modification of carbamidomethyl (C), a variable modifications of oxidation (M) and gly-gly (K) with a peptide tolerance of 25 ppm. Peptide identifications were accepted if they could be established at greater than 95.0% probability and False Discovery Rates (FDRs) based on a random decoy database (which uses the same average amino acid composition and the input sequence). Individual MS/MS were then analysed manually to verify the Mascot matches for the modified peptides.

Circular Dichroism

Circular Dichroism (CD) spectra between 250 and 185 nm were recorded on an AVIV410 spectropolarimeter, at 25°C and 0.5 nm steps. Protein concentrations were determined by UV spectroscopy using theoretical extinction coefficients determined from the amino acids sequence (http://www.expasy.ch/tools/protparam.html). Spectra were measured in 5 mM sodium phosphate pH 7.8, 50 mM NaF, in a 1 mm pathlength quartz cuvette, 1 nm slit-width and 1 second averaging time. Protein samples were centrifuged at 13,000 rpm for 5 minutes at 25°C prior to measurements, to reduce noise due to scattering. The spectra were not corrected for the refractive index of the solvent. For each spectrum the raw data from at least 3 scans was averaged, smoothed, and transformed into mean residue ellipticity ([**θ**]). The transformed data was deconvoluted and analysed by the CDSSTR algorithm (48), using the DichroWeb server (http://dichroweb.cryst.bbk.ac.uk).

Supplementary References

1. L. Aravind, Guilt by association: contextual information in genome analysis. *Genome research* **10**, 1074 (Aug, 2000).
2. M. Y. Galperin, E. V. Koonin, Who's your neighbor? New computational approaches for functional genomics. *Nature biotechnology* **18**, 609 (Jun, 2000).
3. K. S. Makarova, E. V. Koonin, Archaeal ubiquitin-like proteins: functional versatility and putative ancestral involvement in tRNA modification revealed by comparative genomic analysis. *Archaea* **2010**, (2010).
4. S. Gottesman, S. Wickner, M. R. Maurizi, Protein quality control: triage by chaperones and proteases. *Genes Dev* **11**, 815 (Apr 1, 1997).
5. T. Schule, M. Rose, K. D. Entian, M. Thumm, D. H. Wolf, Ubc8p functions in catabolite degradation of fructose-1, 6-bisphosphatase in yeast. *EMBO J* **19**, 2161 (May 15, 2000).
6. A. Karcher, K. Buttner, B. Martens, R. P. Jansen, K. P. Hopfner, X-ray structure of RLI, an essential twin cassette ABC ATPase involved in ribosome biogenesis and HIV capsid assembly. *Structure* **13**, 649 (Apr, 2005).
7. H. V. Miranda *et al.*, E1- and ubiquitin-like proteins provide a direct link between protein conjugation and sulfur transfer in archaea. *Proc Natl Acad Sci U S A* **108**, 4417 (Mar 15, 2011).
8. L. Perrochia, D. Guetta, A. Hecker, P. Forterre, T. Basta, Functional assignment of KEOPS/EKC complex subunits in the biosynthesis of the

universal t6A tRNA modification. *Nucleic Acids Res* **41**, 9484 (Nov 1, 2013).
9. M. Srinivasan *et al.*, The highly conserved KEOPS/EKC complex is essential for a universal tRNA modification, t6A. *EMBO J* **30**, 873 (Mar 2, 2011).
10. L. M. Iyer, K. S. Makarova, E. V. Koonin, L. Aravind, Comparative genomics of the FtsK-HerA superfamily of pumping ATPases: implications for the origins of chromosome segregation, cell division and viral capsid packaging. *Nucleic Acids Res* **32**, 5260 (2004).
11. N. J. Rzechorzek *et al.*, Structure of the hexameric HerA ATPase reveals a mechanism of translocation-coupled DNA-end processing in archaea. *Nature communications* **5**, 5506 (2014).
12. VAST (Vector Alignment Search Tool) at NCBI (http://www.ncbi.nlm.nih.gov/Structure/VAST/vastsearch.html). .
13. L. Holm, P. Rosenstrom, Dali server: conservation mapping in 3D. *Nucleic Acids Res* **38**, W545 (Jul, 2010).
14. DaliLite at EMBL-EBI http://www.ebi.ac.uk/Tools/structure/dalilite/.
15. G. M. Sheldrick, A short history of SHELX. *Acta crystallographica. Section A, Foundations of crystallography* **64**, 112 (Jan, 2008).
16. PROTEUM2, Version 2009 Bruker AXS Inc., Madison, Wisconsin, USA., (2009).
17. APEX2, SAINT-Plus, XPREP and SADABS. Bruker AXS Inc., Madison, Wisconsin, USA., (2004).
18. F. Long, A. A. Vagin, P. Young, G. N. Murshudov, BALBES: a molecular-replacement pipeline. *Acta Crystallogr D Biol Crystallogr* **64**, 125 (Jan, 2008).
19. P. Emsley, B. Lohkamp, W. G. Scott, K. Cowtan, Features and development of Coot. *Acta Crystallogr D Biol Crystallogr* **66**, 486 (Apr, 2010).
20. P. D. Adams *et al.*, PHENIX: a comprehensive Python-based system for macromolecular structure solution. *Acta Crystallogr D Biol Crystallogr* **66**, 213 (Feb, 2010).
21. V. B. Chen *et al.*, MolProbity: all-atom structure validation for macromolecular crystallography. *Acta Crystallogr D Biol Crystallogr* **66**, 12 (Jan, 2010).
22. S. Berkner, A. Wlodkowski, S. V. Albers, G. Lipps, Inducible and constitutive promoters for genetic systems in Sulfolobus acidocaldarius. *Extremophiles* **14**, 249 (May, 2010).
23. M. Wagner *et al.*, Versatile Genetic Tool Box for the Crenarchaeote Sulfolobus acidocaldarius. *Frontiers in microbiology* **3**, 214 (2012).
